# Hydrogen Bonding in Chloro- and Hydroxy-7-Azaindoles: Insights from X-Ray, Vibrational Spectroscopy, and DFT Studies

**DOI:** 10.3390/molecules30234525

**Published:** 2025-11-23

**Authors:** Karolina Dysz, Julia Bąkowicz, Ksenia Szmigiel-Bakalarz, Magdalena Rydz, Barbara Morzyk-Ociepa

**Affiliations:** 1 Military Institute of Engineer Technology, Obornicka 136, 50-961 Wroclaw, Poland; 2 Faculty of Chemistry, Wroclaw University of Science and Technology, Wybrzeże Wyspiańskiego 27, 50-370 Wroclaw, Poland; 3 Institute of Chemistry, Faculty of Science and Technology, Jan Dlugosz University in Czestochowa, Armii Krajowej 13/15, 42-200 Czestochowa, Poland

**Keywords:** 7-azaindole, hydrogen bonding, halogen substituents, hydroxyl substituents, crystal structure, vibrational spectroscopy, DFT calculations

## Abstract

The crystal structures and vibrational spectra of 5-chloro-7-azaindole (5Cl7AI), 4,5-dichloro-7-azaindole (4,5Cl7AI), and 5-hydroxy-7-azaindole (5OH7AI) were investigated to elucidate how ring substituents modulate intermolecular hydrogen bonding and molecular packing in the solid state. Density functional theory (DFT) calculations were employed to support the interpretation of the spectroscopic data, while Hirshfeld surface analysis provided additional insight into intermolecular contacts. Single-crystal X-ray diffraction revealed that the halogenated derivatives form nearly linear N–H···N hydrogen-bonded dimers or layered arrangements, whereas 5OH7AI adopts a three-dimensional network stabilized by N–H···O and O–H···N interactions. FT-IR and FT-Raman spectra showed that variations in hydrogen-bond topology strongly affect the N–H and O–H stretching regions: the halogenated derivatives exhibit broad, red-shifted bands (3300–2500 cm^−1^) characteristic of N–H···N hydrogen bonds, while 5OH7AI displays smaller red shifts of the N–H stretching bands accompanied by some additional features from O–H stretching vibrations. DFT calculations at the B3LYP-D3 and ωB97X-D levels reproduced the experimental geometries and vibrational spectra very well, providing detailed insight into the relationship between hydrogen-bond linearity, network dimensionality, and vibrational behavior.

## 1. Introduction

Hydrogen bonding is one of the most fundamental non-covalent interactions governing molecular organization, stability, and functionality [1,2,3]. In biological systems, it determines the pairing of nucleobases in DNA and the conformational preferences of proteins, while in materials chemistry it controls molecular packing, charge transport, and polymorphism [4,5,6,7,8].

The 7-azaindole (7AI) scaffold has attracted considerable attention due to its structural similarity to purine bases [9] and its ability to form strong, directional N–H···N hydrogen bonds [10]. These features make 7AI a valuable motif in the design of biomimetic systems and pharmaceutical agents [11,12,13]. Azaindole derivatives exhibit a wide range of biological activities, including anticancer, antibacterial, and kinase-inhibitory effects [14,15]. Moreover, they have been investigated for their optoelectronic properties and excited-state proton transfer (ESPT) behavior [16,17,18,19]. 7AI readily forms stable N–H···N hydrogen-bonded dimers, which serve as model systems for studying hydrogen-bond dynamics and tautomeric equilibria [20].

In the solid state, unsubstituted 7AI forms cyclic tetramers [21], whereas halogenated derivatives typically adopt centrosymmetric dimers stabilized by nearly linear N–H···N hydrogen bonds [22,23,24], often accompanied by additional non-covalent interactions such as C–H···X and C–H···π contacts [25,26,27]. However, previous studies have focused primarily on halogenated derivatives. No systematic comparison of the effects of halogen versus hydroxyl substituents (at the C5 position of 7AI) on shaping hydrogen-bond networks and molecular packing has yet been made. Understanding these substituent effects is important for both fundamental science and practical applications: in pharmaceutical chemistry, small changes in hydrogen-bonding patterns can alter molecular recognition and binding affinity, while in materials science they can influence molecular stacking, optical transitions, and charge-transport properties [28,29]. Vibrational spectroscopy is a particularly sensitive tool for studying hydrogen bonding, since shifts in the N–H and O–H stretching frequencies provide direct insight into the strength and geometry of these bonds.

In this paper, we report for the first time the crystal structures of 5-chloro-7-azaindole (5Cl7AI), 4,5-dichloro-7-azaindole (4,5Cl7AI), and 5-hydroxy-7-azaindole (5OH7AI). In 5OH7AI, the hydroxyl group at C5 introduces additional hydrogen-bonding possibilities, leading to a packing motif distinct from that of the halogenated analogues. The compounds were characterized by single-crystal X-ray diffraction, infrared, and Raman spectroscopy. The experimental findings were supported by density functional theory (DFT) calculations used to assist in the assignment of vibrational modes and to confirm the observed hydrogen-bonding motifs.

Unlike previous studies focused solely on halogenated 7AI derivatives [25,26,27], this work provides the first systematic comparison of halogen and hydroxyl substituents at the C5 position, revealing how these functional groups influence the hydrogen-bonding patterns and molecular packing in the solid state. This combined structural and spectroscopic approach offers insights into the design of new crystalline and supramolecular systems with tailored hydrogen-bonding architectures.

## 2. Results and Discussion

### 2.1. Crystal and Molecular Structures of 5Cl7AI, 4,5Cl7AI and 5OH7AI

**Figure 1:**
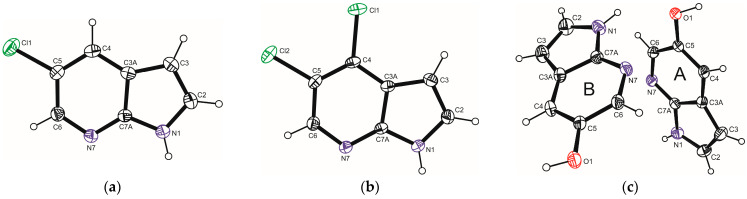
Perspective views of the molecular structures of (**a**) 5Cl7AI, (**b**) 4,5Cl7AI, and (**c**) the two symmetry-independent molecules (A and B) of 5OH7AI, drawn with 25% probability displacement ellipsoids and showing the atom numbering schemes.

The molecular structures of the title compounds are shown in Figure 1.

Crystal data and structure refinement details are summarized in Table S1 (Supplementary Materials). Selected experimental and theoretical bond lengths and bond angles are provided in Table S2 (Supplementary Materials). In all three compounds, the azaindole core remains essentially planar, which is consistent with aromatic conjugation. The experimental bond lengths within the heterocyclic azaindole ring range from 1.331 to 1.427 Å and are very similar to the corresponding theoretical values, which range from 1.328 to 1.435 Å (Table S2). These values indicate delocalized π-conjugation within the ring, in agreement with its essentially planar structure. The C–Cl bond lengths in 5Cl7AI and 4,5Cl7AI range from 1.722 to 1.744 Å (Table S2) and are comparable to those observed in other chloro-substituted azaindoles [23,25,27]. In 5OH7AI, the C–O bond length of approximately 1.366 Å is consistent with values typical for phenolic moieties [30].

**Figure 2:**
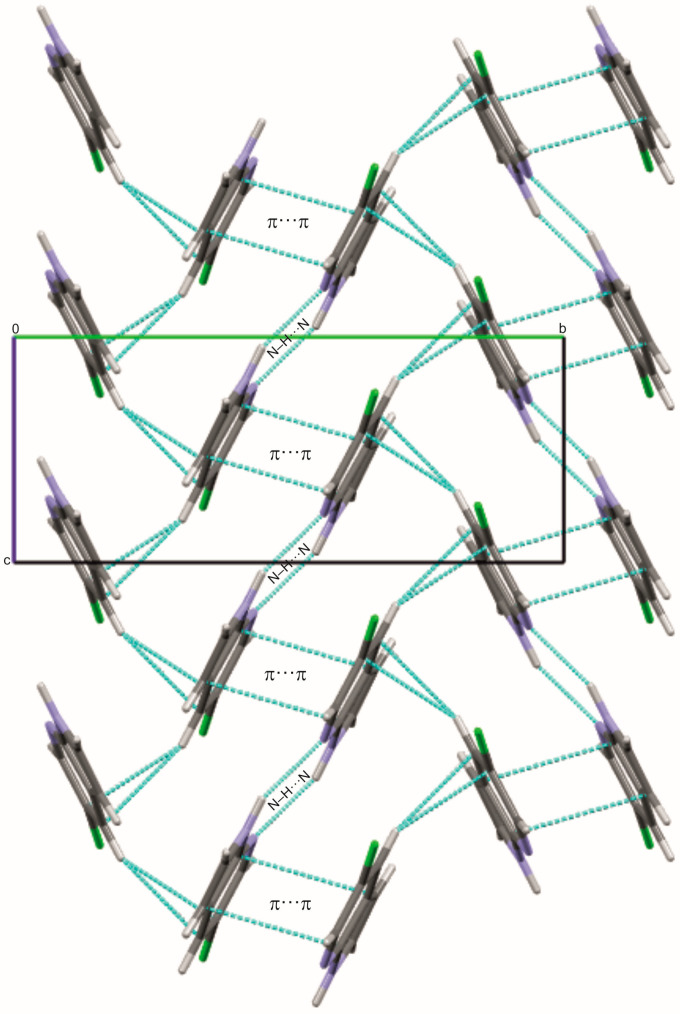
Crystal packing of 5Cl7AI showing the formation of centrosymmetric R^2^_2_(8) N–H···N hydrogen-bonded dimers and π···π stacking interactions (cyan). Atoms are colored as follows: carbon—gray, nitrogen—blue, chlorine—green, and hydrogen—white.

5Cl7AI crystallizes in the monoclinic space group *P*2_1_/*c*, with one molecule in the asymmetric unit. The molecules form centrosymmetric R^2^_2_(8) hydrogen-bonded dimers through N1–H1···N7 interactions, with an N···N distance of 2.983(2) Å and a bond angle of 159.6° (Figure 2; Table S3, Supplementary Materials), representing the fundamental supramolecular building blocks of the packing. These dimers are further stabilized by a C4–H4···π interaction involving the centroid of a neighboring aromatic ring (H···Cg1 = 2.778 Å; ∠H–C–Cg1 = 134.1°), contributing to directional packing along the *b*-axis.

The π···π stacking interactions between adjacent azaindole rings are inferred from the crystal geometry, with an interplanar distance of 3.418 Å, a centroid–centroid separation of 3.734 Å, and a centroid slippage of 1.501 Å (Table S3). Complementary red and blue triangular regions on the Hirshfeld surface mapped over the shape index (Figure S1, Supplementary Materials) support the presence of these π···π contacts.

The combined N–H···N hydrogen bonds and π···π interactions result in a zigzag arrangement of molecules along the *c*-axis, facilitating directional propagation of the supramolecular network. The Hirshfeld surface (Figure S2, Supplementary Materials) further highlights the dominance of H···H and H···Cl contacts, emphasizing the role of weak interactions in stabilizing the crystal lattice.

**Figure 3:**
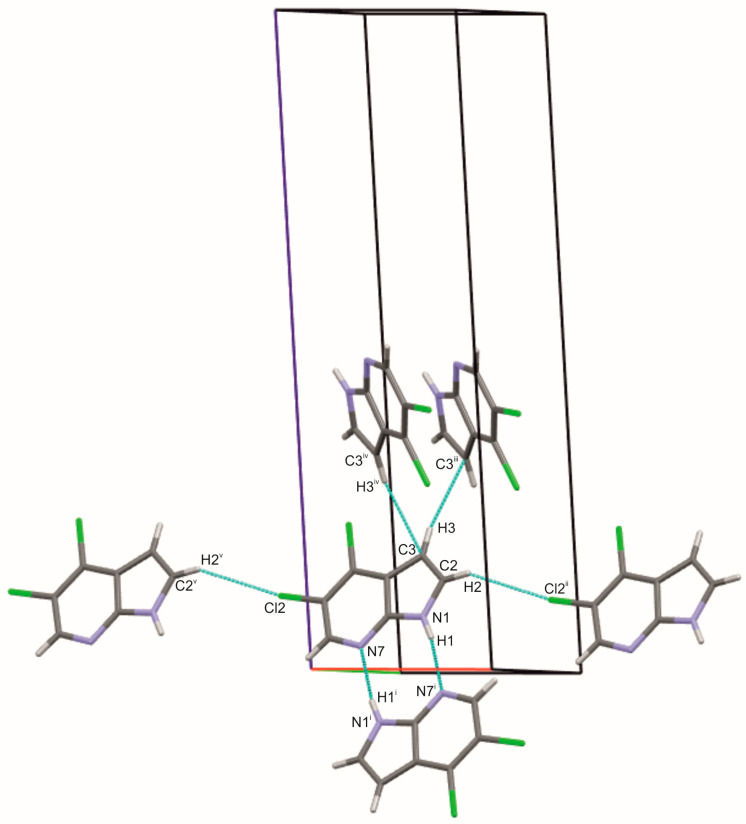
Hydrogen-bonding pattern in the crystal lattice of 4,5Cl7AI, showing the N1–H1···N7, C2–H2···Cl2 and C3–H3···C3 intermolecular interactions (cyan). Symmetry codes: (i) 1 − x, −y, −z; (ii) 1 + x, 1 + y, z; (iii) 1 − x, ½ + y, ½ − z; (iv) 1 − x, −½ + y, ½ − z; (v) −1 + x, −1 + y, z. Atoms are colored as follows: carbon—gray, nitrogen—blue, chlorine—green, and hydrogen—white.

**Figure 4:**
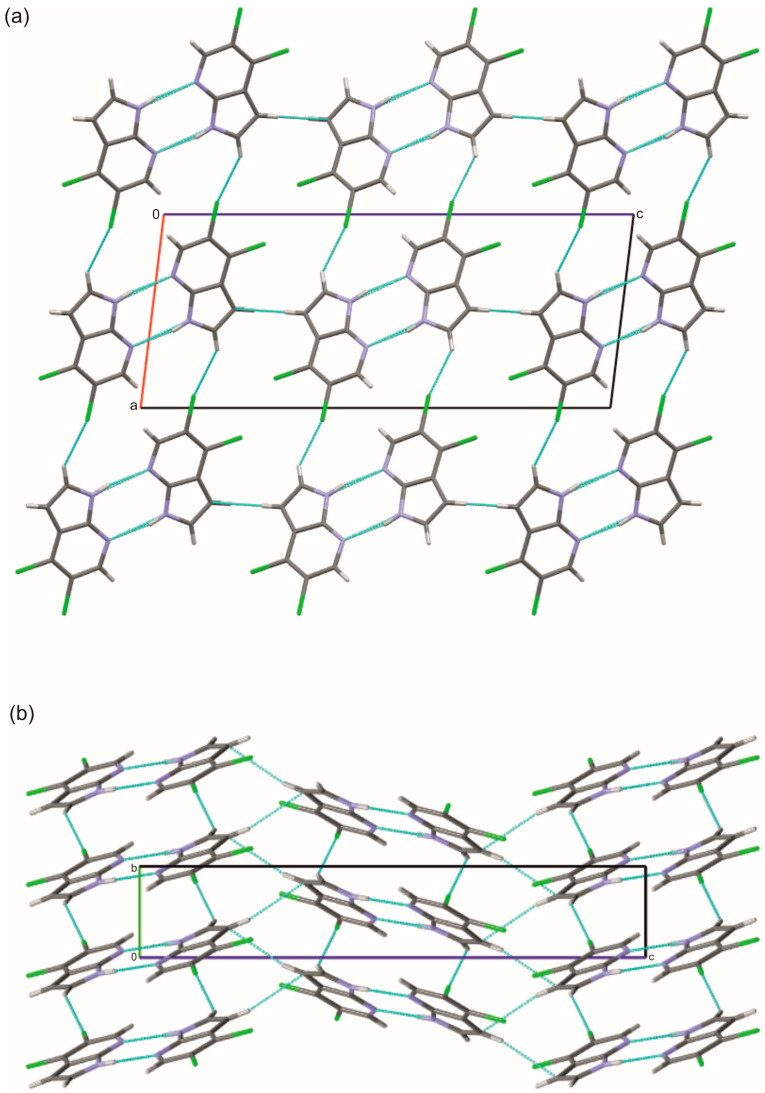
(**a**) View along the *b* axis showing the formation of two-dimensional layers in the crystal structure of 4,5Cl7AI generated by N–H···N, C–H···Cl and C–H···C interactions (cyan); (**b**) View along the *a* axis highlighting the stacking of these hydrogen-bonded layers parallel to the *bc* plane. Atoms are colored as follows: carbon—gray, nitrogen—blue, chlorine—green, and hydrogen—white.

The structure of 4,5Cl7AI, which also crystallizes in the monoclinic space group *P*2_1_/*c*, exhibits a similar primary N–H···N dimeric motif (N···N = 2.939(2) Å). The introduction of a second chlorine substituent at position 4 modifies the molecular packing and gives rise to additional short-range interactions, including C–H···Cl (3.730(2) Å) and C–H···C (3.798(4) Å) contacts, which link the dimers into extended layers parallel to the *bc* plane (Figure 3 and Figure 4; Table S4, Supplementary Materials). The formation of these two-dimensional layers not only enhances packing efficiency but also illustrates how a subtle change in substitution patterns affects the ring geometry and intermolecular interactions. The Cl1 atom at the C4 position influences the local structural parameters of the ring without directly participating in hydrogen bonding, whereas the presence of the Cl2 atom at the C5 position leads to the formation of weak C–H···Cl interactions. These observations indicate that the positions of the Cl substituents modulate the supramolecular topology. The presence of such interactions is further supported by the distribution of contact zones on the Hirshfeld surface (Figure S3, Supplementary Materials).

**Figure 5:**
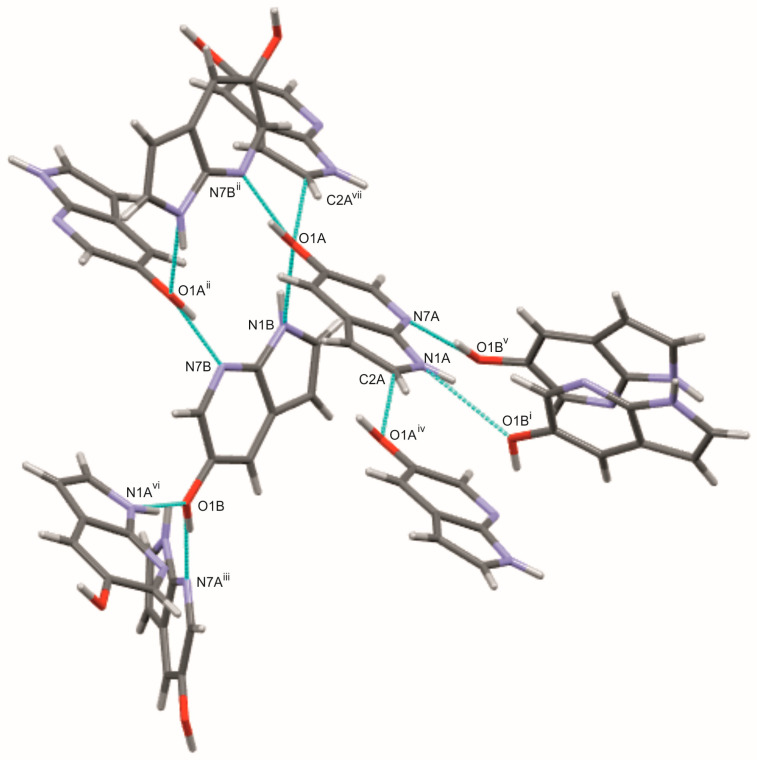
Hydrogen-bonding pattern in the crystal structure of 5OH7AI. View showing the intermolecular N–H···O, O–H···N, and weak C–H···O hydrogen bonds (cyan) connecting two crystallographically independent molecules (A and B) into a three-dimensional network. Symmetry codes: (i) x, 2 − y, −½ + z; (ii) 1 − x, 1 − y, 2 − z; (iii) 3/2 − x, 3/2 − y, ½ + z; (iv) x, 1 + y, z; (v) 3/2 − x, 3/2 − y, −½ + z; (vi) x, 2 − y, ½ + z; (vii) x, −1 + y, z. Atoms are colored as follows: carbon—gray, nitrogen—blue, oxygen—red, and hydrogen—white.

**Figure 6:**
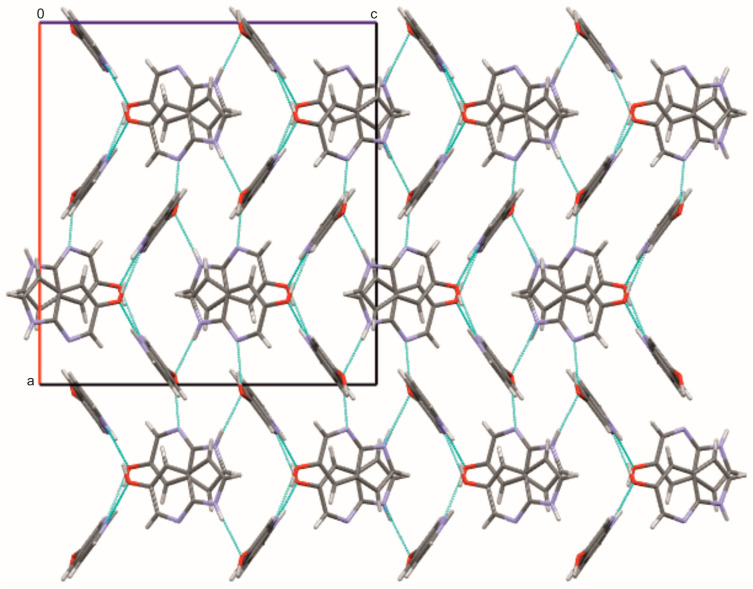
Crystal packing of 5OH7AI viewed along the *b* axis. The extended three-dimensional network is formed through N–H···O and O–H···N hydrogen bonds (cyan), generating layers parallel to the *ac* plane. Atoms are colored as follows: carbon—gray, nitrogen—blue, oxygen—red, and hydrogen—white.

In contrast to the halogenated derivatives, 5OH7AI crystallizes in the orthorhombic space group *Pbcn*, with two independent molecules in the asymmetric unit (Z′ = 2; Figure 1). Replacing the halogen atom with a hydroxyl group results in a markedly different supramolecular arrangement. The hydroxyl group provides opportunities for the formation of additional hydrogen bonds that are not present in the chlorinated analogues, highlighting the critical role of substituent polarity. Molecules are connected through both N–H···O and O–H···N hydrogen bonds, forming an extended three-dimensional network (Figure 5 and Figure 6; Table S5, Supplementary Materials). The O–H···N hydrogen bonds, with donor–acceptor distances of 2.64–2.66 Å and bond angles in the range of 166–172°, are strong and nearly linear, characteristic of classical hydrogen bonds. In contrast, the N–H···O hydrogen bonds (D···A ≈ 2.94 Å, ∠ = 141–152°) are weaker and less linear than the O–H···N hydrogen bonds, and are comparable in strength to the N–H···N hydrogen bonds observed in the chlorinated analogues. A weak C–H···O contact (C2A–H2A···O1A = 3.25 Å) further stabilizes the packing motif.

The –OH substituent, acting as both donor and acceptor, increases the number of possible hydrogen-bonding pathways and promotes the formation of a three-dimensional hydrogen-bonded framework. The coexistence of O–H···N and N–H···O hydrogen bonds introduces asymmetry between the two crystallographically independent molecules (A and B), reflecting slight conformational flexibility within the lattice. This structural diversity gives rise to a more complex hydrogen-bond topology compared with the dimeric or layered arrangements observed in the halogenated analogues. Hirshfeld surface analysis (Figure S4, Supplementary Materials) supports this hydrogen-bonding scheme, showing intense red spots at donor and acceptor regions corresponding to N–H···O and O–H···N intermolecular bonds, as well as weaker C–H···O contacts. These results indicate that the crystal structure of 5OH7AI is dominated by classical hydrogen bonds, while secondary contacts contribute to fine-tuning the overall packing arrangement.

According to Steiner [3], hydrogen bonds in molecular crystals form a continuous spectrum, ranging from classical strong N–H···O and O–H···O interactions to weaker, predominantly electrostatic contacts such as C–H···O, C–H···Cl, and π···π stacking. This concept provides a useful framework for interpreting the results. In 5OH7AI, the short and nearly linear O–H···N hydrogen bonds represent the strong end of this continuum, whereas the less linear N–H···O hydrogen bonds belong to the medium-strength range. This balance exemplifies Desiraju’s concept of cooperative polarization [1], in which strong and weak hydrogen bonds act synergistically to generate a robust, multidimensional supramolecular framework.

The three crystal structures clearly demonstrate how substitution at the C5 position, and additionally at C4 in the dichlorinated compound, modulates the nature and dimensionality of intermolecular interactions. While 5Cl7AI and 4,5Cl7AI adopt dimeric arrangements based on N–H···N hydrogen bonds supplemented by weak contacts such as C–H···π, π···π, and C–H···Cl, the hydroxylated derivative exhibits a highly directional three-dimensional hydrogen-bonded network. This highlights the pronounced influence of substituent polarity on the crystal architecture.

To complement these findings and further elucidate the role of substituents in determining hydrogen-bonding patterns, DFT calculations were performed and compared with the X-ray diffraction data.

### 2.2. DFT Calculations of Molecular Geometry and Intermolecular Interactions

The influence of dual N–H···N hydrogen bonds on the molecular structures of 5Cl7AI and 4,5Cl7AI was examined by means of full geometry optimizations of dimeric models consisting of two molecules connected by the two centrosymmetric N–H···N hydrogen bonds observed in the crystal (see Schemes S1 and S2 of the Supplementary Materials). For 5OH7AI, a tetrameric model containing four molecules, labeled A, A′, B, and B′, was constructed to reproduce the N–H···O and O–H···N hydrogen-bonding network observed in the crystal (see Scheme S3 of the Supplementary Materials). Geometry optimizations were carried out using DFT methods that include dispersion corrections. These are necessary to capture intermolecular interactions and the crystal packing observed experimentally. The optimized dimeric models accurately reproduce the experimental hydrogen-bonding motifs, whereas the tetramer of 5OH7AI retains the hydrogen-bonding pattern but shows a deviation in the relative molecular orientation compared with the crystal structure.

The optimized geometrical parameters have been compared with the X-ray diffraction data in Tables S2–S5 (Supplementary Materials). Calculated bond lengths in the heterocyclic rings show very good agreement with the experimental values, with deviations typically ≤0.02 Å. The C–X bond lengths (where X = Cl or OH) are also well reproduced, although slightly larger deviations are observed for the hydroxyl group in 5OH7AI. Small discrepancies likely arise from the intrinsic mobility of the –OH group within the crystal lattice, the limited accuracy of X-ray diffraction in locating hydrogen atoms, and the constraints of the tetrameric model used in the calculations. Internal bond angles within the heterocyclic rings and those involving the substituents differ from the experimental values by no more than 1° (typically ≤ 1°), confirming the reliability of the computational geometry optimizations.

It should be emphasized that a direct quantitative comparison between the DFT-optimized geometries and experimental SC-XRD structures must be made with caution. DFT calculations yield geometries at the minimum of the potential energy surface of the isolated compound or a cluster of molecules in the gas phase at 0 K, whereas SC-XRD provides time-averaged structures influenced by thermal motion, libration, crystal packing forces, and the intrinsic limitations in locating hydrogen atoms. As discussed in Ref. [31], these fundamental differences imply that small systematic deviations—particularly in hydrogen-bond metrics and intermolecular orientations—are expected. They reflect genuine physical distinctions between theoretical minima and the experimental geometries. Although our cluster models effectively capture the key short-range intermolecular interactions, they approximate the long-range crystal environment only partially. Therefore, the observed minor discrepancies between theoretical and experimental results should be regarded as inherent to such a comparison.

To quantitatively assess the level of agreement between the experimental and theoretical geometries, the average relative deviations (ARDs) were calculated for the bond lengths and bond angles, excluding C–H, N–H, and O–H bonds and the corresponding C–C–H, C–N–H, and C–O–H bond angles. For 5Cl7AI, the ARD values amount to 0.49% for bond lengths and 0.21% for bond angles. For 4,5Cl7AI, the corresponding deviations are 0.57% and 0.35%, respectively. For 5OH7AI, the analysis was performed separately for the two symmetry-independent molecules: for molecule A, the ARDs are 1.01% (bond lengths) and 0.57% (bond angles), while for molecule B they are 0.89% and 0.39%, respectively. Such low ARD values confirm the very good agreement between the optimized and experimental geometries of these molecules and validate the reliability of the applied DFT methods (Table S2 in Supplementary Materials).

Theoretical calculations also reproduce the key intermolecular interactions observed in the crystal structures. For 5Cl7AI and 4,5Cl7AI, the centrosymmetric N–H···N hydrogen bonds are well described, with the calculated bond lengths being slightly shorter and the bond angles slightly more linear than those obtained from the X-ray data (Tables S3 and S4, Supplementary Materials). In the case of 5OH7AI, the N–H···O hydrogen bonds are only marginally shorter than the experimental ones, whereas the O–H···N interactions appear slightly elongated in the DFT calculations. This small deviation likely results from the simplified tetrameric model, which preserves the hydrogen bonds but does not fully capture the long-range packing effects present in the solid state (Table S5, Supplementary Materials).

In summary, the DFT calculations provide a consistent and quantitatively accurate description of both the molecular geometries and the hydrogen-bonding patterns for all three compounds. Good overall agreement between theoretical and experimental data validates the use of the DFT-optimized models for further analyses, including the calculation and interpretation of vibrational spectra.

### 2.3. Influence of Intermolecular Interactions on Vibrational Spectra

**Figure 7:**
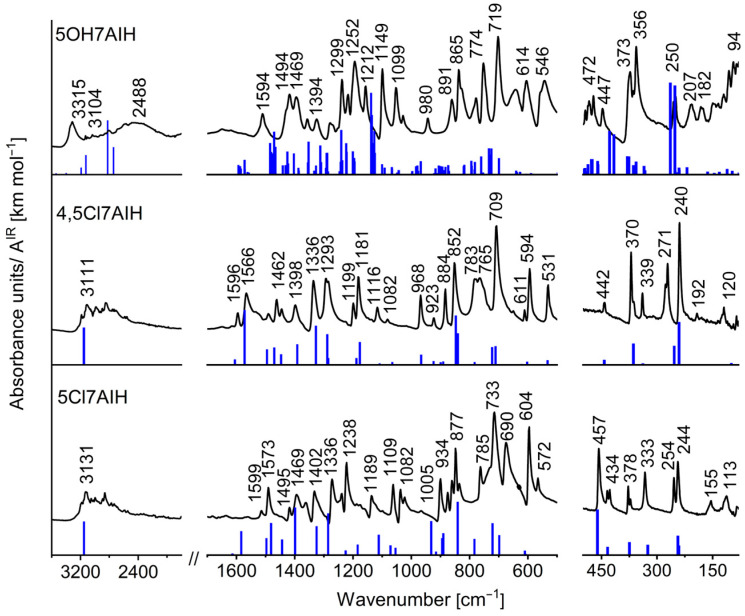
Experimental and theoretical IR spectra of 5Cl7AI, 4,5Cl7AI, and 5OH7AI in the range 3600–80 cm^−1^. The experimental spectra are shown in black. The theoretical spectra were calculated at the ωB97X-D/6-31++G(d,p) level for 5Cl7AI and 4,5Cl7AI, and at the B3LYP-D3/6-31++G(d,p) level for 5OH7AI, and are shown as blue sticks corresponding to the calculated wavenumbers.

**Figure 8:**
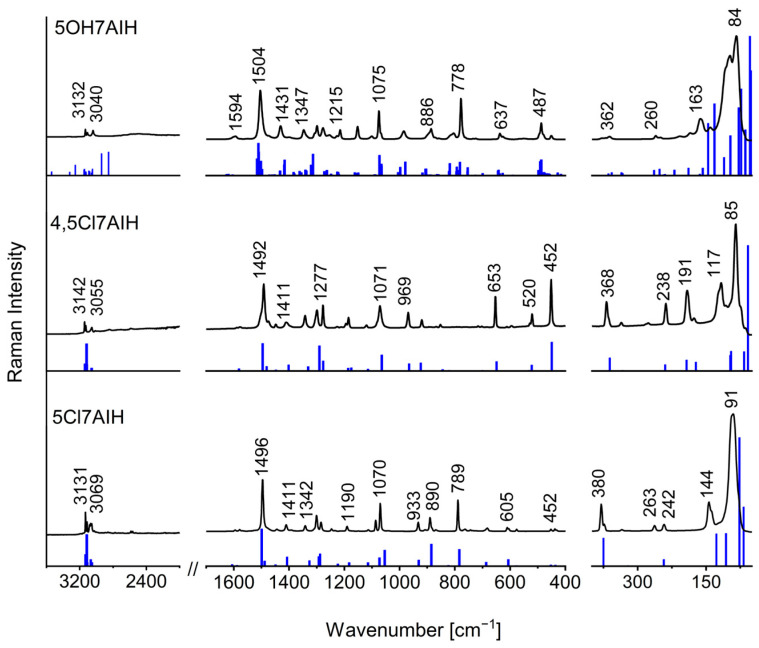
Experimental and theoretical Raman spectra of 5Cl7AI, 4,5Cl7AI, and 5OH7AI in the range 3600–80 cm^−1^. The experimental spectra are shown in black. The theoretical spectra were calculated at the ωB97X-D/6-31++G(d,p) level for 5Cl7AI and 4,5Cl7AI, and at the B3LYP-D3/6-31++G(d,p) level for 5OH7AI, and are shown as blue sticks corresponding to the calculated wavenumbers.

The vibrational spectra of 5Cl7AI, 4,5Cl7AI, and 5OH7AI were analyzed to evaluate the impact of intermolecular hydrogen bonding on the characteristic vibrational bands. The experimental FT-IR and FT-Raman spectra were compared with the theoretical spectra calculated for the optimized structures (Figure 7 and Figure 8; Tables S6–S8, Supplementary Materials). In the case of 5OH7AI, the theoretical spectra include additional blue sticks corresponding to the stretching vibrations of free NH and OH groups. Therefore, these “extra bands” do not have any counterparts in the experimental spectra. As follows from this comparison, a tetrameric model, which includes intermolecular hydrogen bonds between symmetry-independent molecules (A/B and A′/B′), can reproduce the solid-state vibrational spectra of 5OH7AI.

The key geometrical parameters of intermolecular hydrogen bonds and the corresponding N–H/O–H stretching frequencies are summarized in Table 1.

In the N–H stretching region, the two chlorinated derivatives exhibit strong and broad absorption bands with complex substructures extending over the 3300–2500 cm^−1^ range (Figure 7). These spectral features are characteristic of doubly hydrogen-bonded N–H···N dimers. A similar broad, complicated, and red-shifted N–H absorption band was also observed for solid 7-azaindole [32]. Such a multi-component band shape has been attributed to extensive Fermi resonances between the N–H stretching vibrations and overtone or combination bands in dimers, as described by Dreyer [33]. The same spectral patterns were reported for previously studied halogenated 7-azaindoles, including 3-chloro-, 5-bromo-, and other substituted derivatives [25,26,27].

The similarity of the spectral profiles for 5Cl7AI and 4,5Cl7AI demonstrates the presence of two nearly linear N–H···N hydrogen bonds, leading to the formation of dimeric (5Cl7AI) or layered (4,5Cl7AI) supramolecular motifs. The pronounced red shift of the N–H stretching bands from the typical range of free vibrations (~3500 cm^−1^) to 3300–2500 cm^−1^ is consistent with the presence of medium-strength hydrogen bonds, as classified by Steiner [3], further stabilized by weaker C–H···π and C–H···Cl interactions.

In contrast to the chlorinated derivatives, the N–H stretching band of 5OH7AI is observed at a higher frequency (3315 cm^−1^), despite comparable donor–acceptor distances (~2.94 Å). This effect results from a more complex three-dimensional hydrogen-bonding network involving both N–H···O and O–H···N interactions. Unlike the relatively simple dimers or layers in the halogenated derivatives, the hydrogen bonds in 5OH7AI show deviations from linearity and form multiple interconnected pathways.

This geometry reduces the characteristic red shift of the N–H stretching vibrations, whereas the O–H stretching modes appear as a broad band in the 2800–2000 cm^−1^ region, reflecting the strong O–H···N hydrogen bonds, in agreement with Desiraju’s concept of cooperative polarization [1]. This spectral behavior highlights the cooperative nature of hydrogen bonding, where the interplay between donor and acceptor sites leads to collective frequency shifts that cannot be explained by isolated molecular models alone.

A maximum appearing at 2488 cm^−1^ in the experimental FT-IR spectrum of 5OH7AI (Figure 7) does not correspond to a fundamental O–H stretching vibration. Instead, this feature is most plausibly attributed to an overtone or combination band. Such an interpretation is fully consistent with observations in other organic molecular crystals. For example, in the IR spectrum of crystalline menadione (Vitamin K_3_), the 2800–1800 cm^−1^ region is dominated by anharmonic transitions, with combination bands prevailing over first overtones, which appear clearly at 2747, 2655, 2625 and 2008 cm^−1^ [34]. Although direct anharmonic calculations for the tetramer of 5OH7AI are not feasible, the available harmonic DFT frequencies after scaling can reliably support the assignment of the O–H···N stretching modes within the 2800–2000 cm^−1^ region.

For the chlorinated derivatives, scaled harmonic antisymmetric N–H stretching modes (ν(NH_HB_), Aᵤ symmetry) were predicted around 3150 cm^−1^, in good agreement with the broad experimental absorptions observed between 3300 and 2500 cm^−1^. This supports the presence of nearly linear N–H···N hydrogen bonds forming dimeric or layered structures. For 5OH7AI, the calculations reproduced two distinct types of hydroxyl stretching vibrations: ν(OH_F_) near 3684 cm^−1^, corresponding to non-hydrogen-bonded O–H groups, and ν(OH_HB_) around 2939 and 2854 cm^−1^, assigned to strongly hydrogen-bonded O–H groups. The coexistence of N–H···O and O–H···N interactions in the tetrameric structure leads to a smaller red shift of the calculated N–H stretching vibrations (3321 and 3252 cm^−1^) compared with the chlorinated analogues.

In addition to the high-frequency N–H and O–H stretching vibrations, the calculated spectra of the chlorinated derivatives reveal low-frequency intermolecular modes below 100 cm^−1^ (Tables S6 and S7), which correspond to the stretching and bending vibrations of the N–H···N hydrogen-bonded bridges. For 5OH7AI, the analogous modes associated with the N–H···O and O–H···N linkages are predicted at higher frequencies, between approximately 200 and 100 cm^−1^ (Table S8), confirming the presence of multiple cooperative hydrogen bonds in the three-dimensional network.

## 3. Materials and Methods

### 3.1. Preparation of Crystals of 5Cl7AI, 4,5Cl7AI and 5OH7AI

The studied compounds were purchased from AChemBlock (Advanced ChemBlocks Inc., Hayward, CA, USA). Single crystals suitable for X-ray diffraction analysis were obtained by slow evaporation of solutions at room temperature. Specifically, 5Cl7AI crystallized from ethanol, while 4,5Cl7AI and 5OH7AI crystallized from methanol.

### 3.2. X-Ray Diffraction Analysis

X-ray diffraction data were collected using a four-circle diffractometer equipped with a CCD detector [35]. Data collection and reduction were carried out with the CrysAlis PRO software (version 1.171.41.93a; Rigaku Oxford Diffraction Ltd., Yarnton, Oxfordshire, UK). The structures were solved by direct methods using SHELXS- 2013/1 [36] and refined by full-matrix least-squares on F^2^ using SHELXL-2014 [37]. All non-hydrogen atoms were refined anisotropically. The hydroxyl hydrogen atoms were placed in calculated positions using AFIX 148 in SHELXL and refined as rotating riding hydrogens with Uiso(H) = 1.5Ueq(O). The remaining hydrogen atoms were placed in calculated positions and refined using a riding model with fixed bond lengths C–H = 0.93 Å and N–H = 0.86 Å, and with Uiso(H) = 1.2Ueq(C,N). Molecular graphics were generated using ORTEP-3 [38] and Mercury [39]. Hirshfeld surface analysis was performed with CrystalExplorer (version 21), following the methodology described by [40].

Crystallographic data have been deposited with the Cambridge Crystallographic Data Centre under the deposition numbers CCDC 2494228 (5Cl7AI), 2494229 (4,5Cl7AI), and 2494230 (5OH7AI).

### 3.3. Spectroscopic Measurements

Fourier transform mid-infrared (MIR) and far-infrared (FIR) spectra were recorded using a Bruker VERTEX 70V spectrometer (Bruker Optics GmbH & Co. KG, Ettlingen, Germany) equipped with a diamond ATR accessory and an air-cooled DTGS detector. The spectrometer was maintained under vacuum at a pressure below 1 hPa, and spectra were collected at a resolution of 2 cm^−1^, averaging 64 scans per measurement. The ATR spectra were converted from reflectance (R) to absorbance (A) using OPUS™ 7.5 software. This transformation employed a logarithmic relationship, without applying any correction for the thickness of the sample layer.

FT–Raman (FTR) spectra were recorded in the 3500–50 cm^−1^ range using a Bruker MultiRAM spectrometer (Bruker Optics GmbH & Co. KG, Ettlingen, Germany) equipped with a liquid-nitrogen-cooled germanium detector and an Nd:YAG laser operating at 1064 nm. The spectra were obtained at a resolution of 4 cm^−1^, averaging 256 scans per measurement. The laser power was set to 300 mW, and the diameter of the irradiated area on the sample surface was approximately 5 mm.

The original MIR, FIR, and FTR spectra for all studied compounds are provided in Figures S5–S13 in the Supplementary Materials.

### 3.4. Theoretical Methods

All calculations were performed using the Gaussian 16 program package [41]. Geometry optimizations were carried out at the density functional theory (DFT) level using the 6-31++G(d,p) basis set [42,43]. Two functionals were employed, both including Grimme’s D3 dispersion correction [44]: B3LYP-D3, a hybrid functional combining Becke’s three-parameter exchange functional [45] with the Lee–Yang–Parr correlation functional [46], as described by Stephens et al. [47], and ωB97X-D, a long-range-corrected hybrid functional with empirical dispersion [48].

Optimizations of the 5Cl7AI and 4,5Cl7AI dimers using the ωB97X-D functional were successful, yielding vibrational spectra with no imaginary frequencies and confirming that the obtained structures correspond to true minima on the potential energy surface (PES). In contrast, application of the ωB97X-D functional to the 5OH7AI tetramer led to the presence of imaginary frequencies in the calculated spectrum. This indicates that the optimized structure corresponds to a saddle point, not to a true minimum on PES. Therefore, we have used another DFT method, the B3LYP-D3 functional, for optimization of the 5OH7AI tetramer. The spectra calculated for the obtained structure revealed only real frequencies.

Raman intensities were calculated following the procedure described in [49]. Theoretical vibrational frequencies were scaled to improve agreement with experimental data. For B3LYP-D3, scaling factors of 0.961 (≥2000 cm^−1^), 0.979 (1000–2000 cm^−1^), and 1.000 (<1000 cm^−1^) were applied; for ωB97X-D, the corresponding values were 0.948, 0.953, and 0.970, respectively, as recommended in [50]. Potential energy distributions (PEDs) were determined using the FCART program (version 7.0) [51] to support vibrational assignments. Molecular geometries and normal vibrational modes were visualized using Chemcraft (version 1.8) [52]. The symmetry codes for the molecular models of 5Cl7AI, 4,5Cl7AI, and 5OH7AI are provided in the Supplementary Materials.

## 4. Conclusions

The combined crystallographic, spectroscopic, and theoretical study of 5Cl7AI, 4,5Cl7AI, and 5OH7AI reveals for the first time how different substituents at the C5 (and C4) positions in these 7-azaindole derivatives control the dimensionality, strength, and cooperativity of intermolecular hydrogen-bonding networks, thereby affecting the crystal packing and vibrational properties.

The halogenated compounds, 5Cl7AI and 4,5Cl7AI, form nearly linear N–H···N hydrogen-bonded dimers or layered arrangements, with N···N distances of 2.983 (2) Å and 2.939 (2) Å, respectively, as confirmed by single-crystal X-ray diffraction and DFT calculations of dimers. These motifs are reflected in the presence of broad N–H stretching bands (3300–2500 cm^−1^) showing significant red shifts, characteristic of medium-strength hydrogen bonds.

In 5OH7AI, the hydroxyl substituent promotes the formation of a three-dimensional network, characterized by a strong, nearly linear O–H···N hydrogen bond (D···A = 2.64–2.66 Å, ∠ = 166–175°) and weaker, less linear N–H···O hydrogen-bonding interactions (D···A ≈ 2.94 Å, ∠ = 141–152°). This heterogeneity gives rise to a complex vibrational pattern: the N–H stretching bands exhibit moderate red shifts compared with the chlorinated analogues, while the strongly hydrogen-bonded O–H groups produce broad absorptions in the 2800–2000 cm^−1^ region, consistent with cooperative polarization effects within the network.

DFT calculations accurately reproduce both molecular geometries and intermolecular interactions, with average relative deviations of ≤1.01% for bond lengths and ≤0.57% for bond angles. The optimized molecular models effectively capture the essential hydrogen-bonding patterns observed experimentally, which validates the reliability of the DFT methods (B3LYP-D3 and ωB97X-D) used in these computations.

These results demonstrate that the type of substituent and its position on the 7AI ring directly affect the dimensionality, strength, and cooperativity of the hydrogen-bond networks, which in turn govern supramolecular packing and vibrational properties. The study provides detailed, structure-specific insights that can guide the rational design of functional materials and bioactive compounds based on 7-azaindole derivatives.

## Figures and Tables

**Table 1:** Geometrical parameters of the key intermolecular hydrogen bonds and the corresponding N–H/O–H stretching frequencies for 5Cl7AI, 4,5Cl7AI and 5OH7AI.

Compound	H-Bond Type	H···A (Å)	D···A (Å)	D···A (Å)	D–H···A (°)	ν(N–H/O–H) (cm^−1^)	Network
5Cl7AI	N1–H1···N7	0.86	2.16	2.983 (2)	160	3300–2500	Dimer
4,5Cl7AI	N1–H1···N7	0.86	2.09	2.939 (2)	169	3300–2500	Layer
5OH7AI (A)	N1A–H1A···O1B	0.86	2.15	2.938 (2)	152	3315	3D
5OH7AI (B)	N1B–H1B···O1A	0.86	2.23	2.941 (2)	141	3315	3D
5OH7AI (A)	O1A–H11A···N7B	0.93 (2)	1.73 (2)	2.643 (2)	166	2800–2000	3D
5OH7AI (B)	O1B–H11B···N7A	0.88 (3)	1.79 (3)	2.663 (2)	172	2800–2000	3D

Notes: D = donor atom, A = acceptor atom; ν(N–H/O–H) values are from FT-IR spectra; geometrical parameters from single-crystal X-ray diffraction; network type indicates supramolecular arrangement; (A) and (B) denote different molecules in the asymmetric unit.

## Data Availability

The original contributions presented in this study are included in the article or Supplementary Materials, and further inquiries can be directed to the corresponding author.

## Supplementary Materials

The following supporting information can be downloaded at: https://www.mdpi.com/article/10.3390/molecules30234525/s1, Table S1: Crystal data and structure refinement for 5Cl7AI, 4,5Cl7AI and 5OH7AI; Scheme S1: Optimized geometry of 5Cl7AI dimer obtained at the ωB97X-D/6-31++G(d,p) level; Scheme S2: Optimized geometry of 4,5Cl7AI dimer obtained at the ωB97X-D/6-31++G(d,p) level; Scheme S3: Optimized geometry of 5OH7AI tetramer obtained at the B3LYP-D3/6-31++G(d,p) level; Table S2: Selected experimental bond lengths and bond angles (X-ray) together with theoretical values calculated at the ωB97X-D/6-31++G(d,p) level for 5Cl7AIH and 4,5Cl7AIH, and at the B3LYP-D3/6-31++G(d,p) level for 5OH7AIH. Standard uncertainties (s.u.) are given in parentheses; Table S3: Geometrical parameters of intermolecular interactions (Å, °) in the crystal structure of 5Cl7AI as determined by X-ray diffraction, and the corresponding theoretical values calculated at the ωB97X-D/6-31++G(d,p) level. Standard uncertainties (s.u.) for the experimental data are given in parentheses; Table S4: Geometrical parameters of intermolecular interactions (Å, °) in the crystal structure of 4,5Cl7AI as determined by X-ray diffraction, and the corresponding theoretical values calculated at the ωB97X-D/6-31++G(d,p) level. Standard uncertainties (s.u.) for the experimental data are given in parentheses; Table S5: Geometrical parameters of intermolecular interactions (Å, °) in the crystal structure of 5OH7AI as determined by X-ray diffraction, and the corresponding theoretical values calculated at the B3LYP-D3/6-31++G(d,p) level. Standard uncertainties (s.u.) for the experimental data are given in parentheses; Figure S1: Hirshfeld surface of 5Cl7AI mapped over the shape index, showing complementary red and blue triangular regions that indicate π···π stacking interactions between aromatic rings; Figure S2: Red regions on the Hirshfeld surface of 5Cl7AI mapped over *d*_norm_ indicate N–H···N hydrogen bonds and H···H/H···Cl contacts stabilizing the crystal packing; Figure S3: Red regions on the Hirshfeld surface of 4,5Cl7AI mapped over *d*_norm_ indicate N–H···N hydrogen bonds and weaker C–H···Cl and C–H···C contacts stabilizing the crystal packing; Figure S4: Red regions on the Hirshfeld surfaces of 5OH7AI (molecules A and B) mapped over *d*_norm_ indicate N–H···O, O–H···N, and weak C–H···O hydrogen bonds stabilizing the packing; Table S6: Experimental (FT-IR and FT-Raman) and calculated wavenumbers ($\tilde{\nu }$^a^, cm^−1^), infrared intensities (A^IR^, km·mol^−1^), and Raman scattering activities (S^R^, Å^4^·amu^−1^) for 5Cl7AIH obtained at the ωB97X-D/6-31++G(d,p) level; Table S7: Experimental (FT-IR and FT-Raman) and calculated wavenumbers ($\tilde{\nu }$^a^, cm^−1^), infrared intensities (A^IR^, km·mol^−1^), and Raman scattering activities (S^R^, Å^4^·amu^−1^) for 4,5Cl7AIH obtained at the ωB97X-D/6-31++G(d,p) level; Table S8: Experimental (FT-IR and FT–Raman) and calculated wavenumbers ($\tilde{\nu }$^a^, cm^−1^), infrared intensities (A^IR^, km·mol^−1^), and Raman scattering activities (S^R^, Å^4^·amu^−1^) for 5OH7AIH obtained at the B3LYP-D3/6-31++G(d,p) level; Figure S5: Original FT-IR spectrum of 5Cl7AI in the range from 4000 cm^−1^ to 400 cm^−1^; Figure S6: Original FT-IR spectrum of 5Cl7AI in the range from 600 cm^−1^ to 50 cm^−1^; Figure S7: Original FT–Raman spectrum of 5Cl7AI in the range from 3600 cm^−1^ to 50 cm^−1^; Figure S8: Original FT-IR spectrum of 4,5Cl7AI in the range from 4000 cm^−1^ to 400 cm^−1^; Figure S9: Original FT-IR spectrum of 4,5Cl7AI in the range from 600 cm^−1^ to 50 cm^−1^; Figure S10: Original FT-Raman spectrum of 4,5Cl7AI in the range from 3600 cm^−1^ to 50 cm^−1^; Figure S11: Original FT-IR spectrum of 5OH7AI in the range from 4000 cm^−1^ to 400 cm^−1^; Figure S12: Original FT-IR spectrum of 5OH7AI in the range from 600 cm^−1^ to 50 cm^−1^; Figure S13: Original FT-Raman spectrum of 5OH7AI in the range from 3600 cm^−1^ to 50 cm^−1^; Symmetry codes for the molecular models of 5Cl7AI, 4,5Cl7AI and 5OH7AI; Checkcif_5Cl7AI for 5Cl7AI; Checkcif_4,5Cl7AI for 4,5Cl7AI; Checkcif_5OH7AI for 5OH7AI; CCDC 2494228 contains the supplementary crystallographic data for 5Cl7AI, CCDC 2494229 contains the supplementary crystallographic data for 4,5Cl7AI, and CCDC 2494230 contains the supplementary crystallographic data for 5OH7AI. These data can also be obtained free of charge via https://www.ccdc.cam.ac.uk/structures/ (accessed on 13 October 2025) or from the Cambridge Crystallographic Data center, 12 Union Road, Cambridge CB2 1EZ, UK: fax (+44)-1223-336-033 or e-mail deposit@ccdc.cam.ac.uk.
