# Supplementary material for: Hydrogen Bonding in Chloro- and Hydroxy-7-Azaindoles: Insights from X-Ray, Vibrational Spectroscopy, and DFT Studies
Source: Molecules. 2025 Nov 23;30(23):4525. doi: 10.3390/molecules30234525 (PMC12692938; doi:10.3390/molecules30234525)

checkCIF/PLATON report

Structure factors have been supplied for datablock(s) 5Cl7AIH_2

THIS REPORT IS FOR GUIDANCE ONLY. IF USED AS PART OF A REVIEW PROCEDURE FOR PUBLICATION, IT SHOULD NOT REPLACE THE EXPERTISE OF AN EXPERIENCED CRYSTALLOGRAPHIC REFEREE.

No syntax errors found.   CIF dictionary   Interpreting this report

Datablock: 5Cl7AIH_2

```
Bond precision:   C-C = 0.0028 A           Wavelength=0.71073

Cell:             a=8.8127(2)     b=13.6824(4)        c=5.79007(19)
                  alpha=90        beta=104.748(3)     gamma=90
Temperature:      298 K
```

	Calculated	Reported
Volume	675.16(3)	675.16(3)
Space group	P 21/c	P 21/c
Hall group	-P 2ybc	-P 2ybc
Moiety formula	C7 H5 Cl N2	?
Sum formula	C7 H5 Cl N2	C7 H5 Cl N2
Mr	152.58	152.58
Dx,g cm-3	1.501	1.501
Z	4	4
Mu (mm-1)	0.474	0.474
F000	312.0	312.0
F000'	312.67	
h,k,lmax	10,16,7	10,16,7
Nref	1329	1329
Tmin,Tmax	0.903,0.972	0.990,1.000
Tmin'	0.901	

```
Correction method= # Reported T Limits: Tmin=0.990 Tmax=1.000
AbsCorr = MULTI-SCAN
```

```
Data completeness= 1.000          Theta(max)= 25.998

R(reflections)= 0.0416( 1078)     wR2(reflections)= 0.1110( 1329)

S = 1.063                 Npar= 91
```

```
The following ALERTS were generated. Each ALERT has the format
       test-name_ALERT_alert-type_alert-level.
Click on the hyperlinks for more details of the test.
```

Alert level C

```
PLAT906_ALERT_3_C Large K Value in the Analysis of Variance ......      3.533 Check
PLAT918_ALERT_3_C Reflection(s) with I(obs) much Smaller I(calc) .          1 Check
                0  4  0,
```

Alert level G

```
PLAT007_ALERT_5_G Number of Unrefined Donor-H Atoms .............          1 Report
              H1
PLAT941_ALERT_3_G Average HKL Measurement Multiplicity ..........        1.8 Low
PLAT967_ALERT_5_G Note: Two-Theta Cutoff Value in Embedded .res ..      52.0 Degree
PLAT969_ALERT_5_G The 'Henn et al.' R-Factor-gap value ..........      5.483 Note
              Predicted wR2: Based on SigI**2  2.02 or SHELX Weight 10.45
PLAT978_ALERT_2_G Number C-C Bonds with Positive Residual Density.          0 Info
```

```
   0 ALERT level A = Most likely a serious problem - resolve or explain
   0 ALERT level B = A potentially serious problem, consider carefully
   2 ALERT level C = Check. Ensure it is not caused by an omission or oversight
   5 ALERT level G = General information/check it is not something unexpected

   0 ALERT type 1 CIF construction/syntax error, inconsistent or missing data
   1 ALERT type 2 Indicator that the structure model may be wrong or deficient
   3 ALERT type 3 Indicator that the structure quality may be low
   0 ALERT type 4 Improvement, methodology, query or suggestion
   3 ALERT type 5 Informative message, check
```

It is advisable to attempt to resolve as many as possible of the alerts in all categories. Often the minor alerts point to easily fixed oversights, errors and omissions in your CIF or refinement strategy, so attention to these fine details can be worthwhile. It is up to the individual to critically assess their own results and, if necessary, seek expert advice.

PLATON version of 26/09/2025; check.def file version of 20/09/2025

---

duplicate check

No duplication found

Datablock 5Cl7AIH_2 - ellipsoid plot

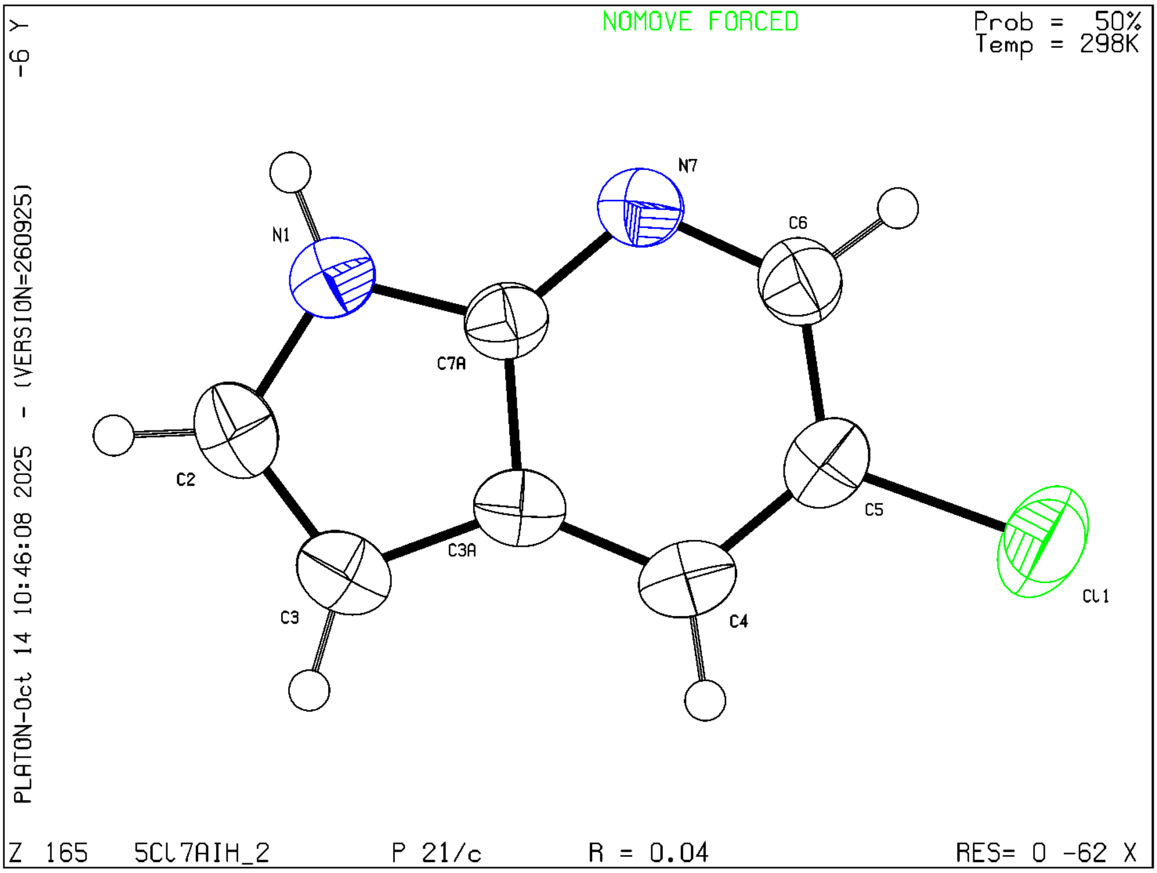

Supplement: Supplementary file 1 [file molecules-30-04525-s001.zip › Checkcif_5Cl7AI.pdf]
