# Supplementary material for: Hydrogen Bonding in Chloro- and Hydroxy-7-Azaindoles: Insights from X-Ray, Vibrational Spectroscopy, and DFT Studies
Source: Molecules. 2025 Nov 23;30(23):4525. doi: 10.3390/molecules30234525 (PMC12692938; doi:10.3390/molecules30234525)

checkCIF/PLATON report

Structure factors have been supplied for datablock(s) 5OH7AIH

THIS REPORT IS FOR GUIDANCE ONLY. IF USED AS PART OF A REVIEW PROCEDURE FOR PUBLICATION, IT SHOULD NOT REPLACE THE EXPERTISE OF AN EXPERIENCED CRYSTALLOGRAPHIC REFEREE.

No syntax errors found. CIF dictionary Interpreting this report

Datablock: 5OH7AIH

Bond precision:	C-C = 0.0022 A		Wavelength=0.71073
Cell:	a=17.7750(4)	b=8.7572(2)	c=16.5601(5)
	alpha=90	beta=90	gamma=90
Temperature:	298 K		

	Calculated	Reported
Volume	2577.73(11)	2577.73(11)
Space group	P b c n	P b c n
Hall group	-P 2n 2ab	-P 2n 2ab
Moiety formula	C7 H6 N2 O	?
Sum formula	C7 H6 N2 O	C14 H12 N4 O2
Mr	134.14	268.28
Dx,g cm-3	1.383	1.383
Z	16	8
Mu (mm-1)	0.097	0.097
F000	1120.0	1120.0
F000’	1120.46	
h,k,lmax	21,10,20	21,10,20
Nref	2535	2532
Tmin,Tmax	0.975,0.983	0.900,1.000
Tmin’	0.943	

Correction method= # Reported T Limits: Tmin=0.900 Tmax=1.000
AbsCorr = MULTI-SCAN

```
Data completeness= 0.999            Theta(max)= 25.995

                                                      wR2(reflections)=
R(reflections)= 0.0405( 2051)                         0.1106( 2532)

S = 1.038                  Npar= 185
```

```
The following ALERTS were generated. Each ALERT has the format
       test-name_ALERT_alert-type_alert-level.
Click on the hyperlinks for more details of the test.
```

Alert level C

```
PLAT041_ALERT_1_C Calc. and Reported SumFormula    Strings  Differ     Please Check
              Calc: C7 H6 N2 O
              Rep.: C14 H12 N4 O2
PLAT906_ALERT_3_C Large K Value in the Analysis of Variance ......      4.055 Check
PLAT911_ALERT_3_C Missing FCF Refl Between Thmin & STh/L=    0.600          3 Report
                5  3  3,  14  6  4,  13  6  5,
```

Alert level G

```
PLAT007_ALERT_5_G Number of Unrefined Donor-H Atoms .............          2 Report
              H1A     H1B
PLAT045_ALERT_1_G Calculated and Reported Z Differ by a Factor ...          2 Check
PLAT720_ALERT_4_G Number of Unusual/Non-Standard Labels .........          4 Note
              C3AA    C7AA    C3AB    C7AB
PLAT941_ALERT_3_G Average HKL Measurement Multiplicity ..........        2.2 Low
PLAT967_ALERT_5_G Note: Two-Theta Cutoff Value in Embedded .res ..       52.0 Degree
PLAT969_ALERT_5_G The 'Henn et al.' R-Factor-gap value ..........      5.794 Note
              Predicted wR2: Based on SigI**2  1.91 or SHELX Weight 10.66
PLAT978_ALERT_2_G Number C-C Bonds with Positive Residual Density.          1 Info
```

```
   0 ALERT level A = Most likely a serious problem - resolve or explain
   0 ALERT level B = A potentially serious problem, consider carefully
   3 ALERT level C = Check. Ensure it is not caused by an omission or oversight
   7 ALERT level G = General information/check it is not something unexpected

   2 ALERT type 1 CIF construction/syntax error, inconsistent or missing data
   1 ALERT type 2 Indicator that the structure model may be wrong or deficient
   3 ALERT type 3 Indicator that the structure quality may be low
   1 ALERT type 4 Improvement, methodology, query or suggestion
   3 ALERT type 5 Informative message, check
```

It is advisable to attempt to resolve as many as possible of the alerts in all categories. Often the minor alerts point to easily fixed oversights, errors and omissions in your CIF or refinement strategy, so attention to these fine details can be worthwhile. It is up to the individual to critically assess their own results and, if necessary, seek expert advice.

PLATON version of 26/09/2025; check.def file version of 20/09/2025

duplicate check

No duplication found

Datablock 5OH7AIH - ellipsoid plot

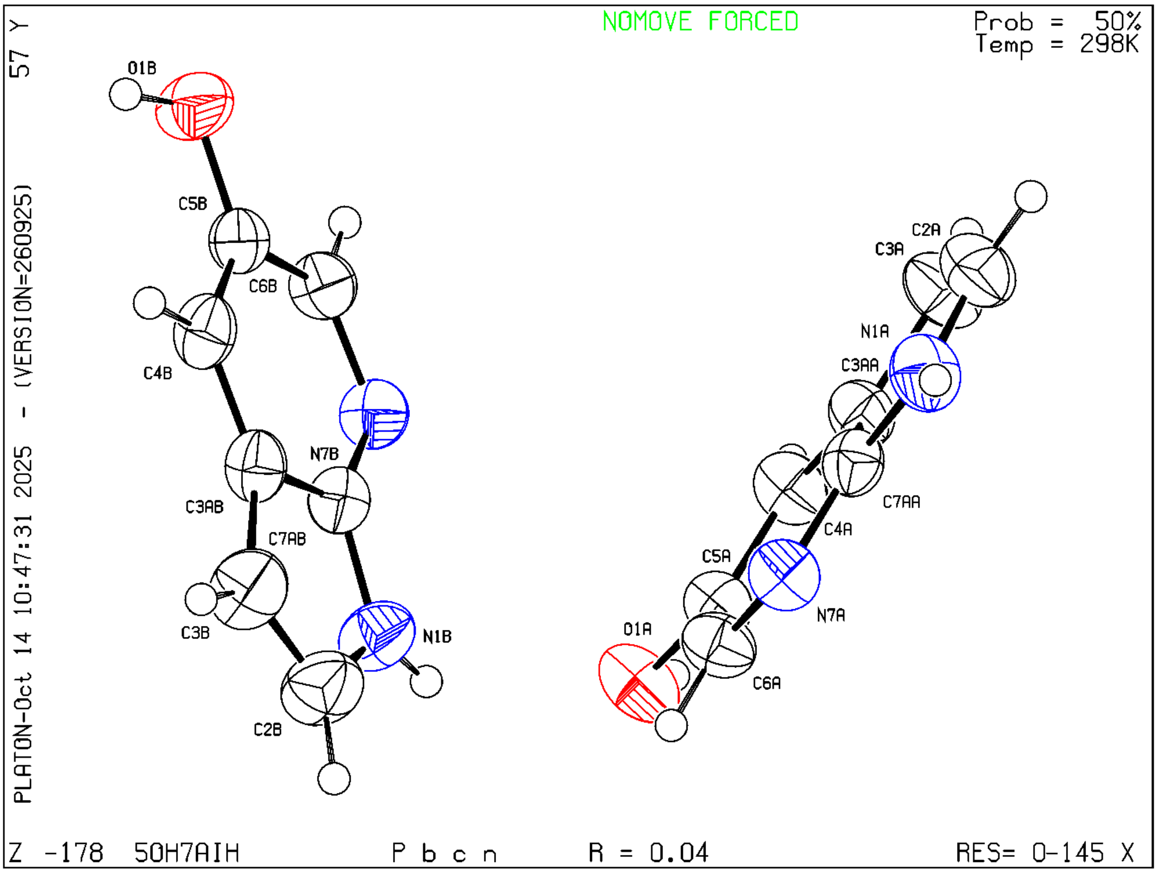

Supplement: Supplementary file 1 [file molecules-30-04525-s001.zip › Checkcif_5OH7AI.pdf]
