# Supplementary material for: Hydrogen Bonding in Chloro- and Hydroxy-7-Azaindoles: Insights from X-Ray, Vibrational Spectroscopy, and DFT Studies
Source: Molecules. 2025 Nov 23;30(23):4525. doi: 10.3390/molecules30234525 (PMC12692938; doi:10.3390/molecules30234525)
Supplement: Supplementary file 1 [file molecules-30-04525-s001.zip › Supplementary Materials_MoleculesR1.pdf]

Supplementary Materials

Hydrogen Bonding in Chloro- and Hydroxy-7-Azaindoles: Insights from X-ray, Vibrational Spectroscopy, and DFT Studies

Karolina Dysz[a], Julia Bąkowicz[b], Ksenia Szmigiel-Bakalarz[c], Magdalena Rydz[b] and Barbara Morzyk-Ociepa[c*]

[a] Military Institute of Engineer Technology, Obornicka 136, 50-961 Wroclaw, Poland,

[b] Faculty of Chemistry, Wroclaw University of Science and Technology, Wybrzeże Wyspiańskiego 27, 50-370 Wroclaw, Poland.

[c] Institute of Chemistry, Faculty of Science and Technology, Jan Dlugosz University in Czestochowa, Armii Krajowej 13/15, 42-200 Czestochowa, Poland,

Keywords: 7-azaindole, hydrogen bonding, halogen substituents, hydroxyl substituents, crystal structure, vibrational spectroscopy, DFT calculations

* Correspondence email: bmorzyk-ociepa@o2.pl

Contents

Table S1. Crystal data and structure refinement for 5Cl7AI, 4,5Cl7AI and 5OH7AI.

Scheme S1. Optimized geometry of 5Cl7AI dimer obtained at the ωB97X-D/6-31++G(d,p) level.

Scheme S2. Optimized geometry of 4,5Cl7AI dimer obtained at the ωB97X-D/6-31++G(d,p) level.

Scheme S3. Optimized geometry of 5OH7AI tetramer obtained at the B3LYP-D3/6-31++G(d,p) level.

Table S2. Selected experimental bond lengths and bond angles (X-ray) together with theoretical values calculated at the ωB97X-D/6-31++G(d,p) level for 5Cl7AIH and 4,5Cl7AIH, and at the B3LYP-D3/6-31++G(d,p) level for 5OH7AIH. Standard uncertainties (s.u.) are given in parentheses.

Table S3. Geometrical parameters of intermolecular interactions (Å, °) in the crystal structure of 5Cl7AI as determined by X-ray diffraction, and the corresponding theoretical values calculated at the ωB97X-D/6-31++G(d,p) level. Standard uncertainties (s.u.) for the experimental data are given in parentheses.

Table S4. Geometrical parameters of intermolecular interactions (Å, °) in the crystal structure of 4,5Cl7AI as determined by X-ray diffraction, and the corresponding theoretical values calculated at the ωB97X-D/6-31++G(d,p) level. Standard uncertainties (s.u.) for the experimental data are given in parentheses.

Table S5. Geometrical parameters of intermolecular interactions (Å, °) in the crystal structure of 5OH7AI as determined by X-ray diffraction, and the corresponding theoretical values calculated at the B3LYP-D3/6-31++G(d,p) level. Standard uncertainties (s.u.) for the experimental data are given in parentheses.

Figure S1. Hirshfeld surface of 5Cl7AI mapped over the shape index, showing complementary red and blue triangular regions that indicate π···π stacking interactions between aromatic rings.

Figure S2. Red regions on the Hirshfeld surface of 5Cl7AI mapped over d_{norm} indicate N–H···N hydrogen bonds and H···H/H···Cl contacts stabilizing the crystal packing.

Figure S3. Red regions on the Hirshfeld surface of 4,5Cl7AI mapped over d_{norm} indicate N–H···N hydrogen bonds and weaker C–H···Cl and C–H···C contacts stabilizing the crystal packing.

Figure S4. Red regions on the Hirshfeld surfaces of 5OH7AI (molecules A and B) mapped over d_{norm} indicate N–H···O, O–H···N, and weak C–H···O hydrogen bonds stabilizing the packing.

Table S6. Experimental (FT-IR and FT-Raman) and calculated wavenumbers (ṽ, cm^{-1}), infrared intensities (A^{IR}, $km·mol^{-1}$), and Raman scattering activities (S^R, $Å^4·amu^{-1}$) for 5Cl7AIH obtained at the ωB97X-D/6-31++G(d,p) level.

Table S7. Experimental (FT-IR and FT-Raman) and calculated wavenumbers (ṽ, cm^{-1}), infrared intensities (A^{IR}, $km·mol^{-1}$), and Raman scattering activities (S^R, $Å^4·amu^{-1}$) for 4,5Cl7AIH obtained at the ωB97X-D/6-31++G(d,p) level.

Table S8. Experimental (FT-IR and FT-Raman) and calculated wavenumbers (ṽ, cm^{-1}), infrared intensities (A^{IR}, $km·mol^{-1}$), and Raman scattering activities (S^R, $Å^4·amu^{-1}$) for 5OH7AIH obtained at the B3LYP-D3/6-31++G(d,p) level.

Figure S5. Original FT-IR spectrum of 5Cl7AI in the range from 4000 cm^{-1} to 400 cm^{-1}.

Figure S6. Original FT-IR spectrum of 5Cl7AI in the range from 600 cm^{-1} to 50 cm^{-1}.

Figure S7. Original FT-Raman spectrum of 5Cl7AI in the range from 3600 cm^{-1} to 50 cm^{-1}.

Figure S8. Original FT-IR spectrum of 4,5Cl7AI in the range from 4000 cm^{-1} to 400 cm^{-1}.

Figure S9. Original FT-IR spectrum of 4,5Cl7AI in the range from 600 cm^{-1} to 50 cm^{-1}.

Figure S10. Original FT-Raman spectrum of 4,5Cl7AI in the range from 3600 cm^{-1} to 50 cm^{-1}.

Figure S11. Original FT-IR spectrum of 5OH7AI in the range from 4000 cm^{-1} to 400 cm^{-1}.

Figure S12. Original FT-IR spectrum of 5OH7AI in the range from 600 cm^{-1} to 50 cm^{-1}.

Figure S13. Original FT-Raman spectrum of 5OH7AI in the range from 3600 cm^{-1} to 50 cm^{-1}.

Symmetry codes for the molecular model of 5Cl7AI.

Symmetry codes for the molecular model of 4,5Cl7AI.

Symmetry codes for the molecular model of 5OH7AI.

Table S1. Crystal data and structure refinement for *5Cl7AI*, *4,5Cl7AI* and *5OH7AI*.

	**5Cl7AI**	**4,5Cl7AI**	**5OH7AI**
Empirical formula	$C_7H_5N_2Cl$	$C_7H_4N_2Cl_2$	$C_{14}H_{12}N_4O_2$
Formula weight	152.58	187.02	268.28
Temperature (K)	298(2)	298(2)	298(2)
Crystal system	Monoclinic	Monoclinic	Orthorhombic
Space group	$P2_1/c$	$P2_1/c$	*Pbcn*
Unit cell dimensions			
a (Å)	8.8127(2)	8.9845(3)	17.7750(4)
b (Å)	13.6824(4)	3.86134(14)	8.7572(2)
c (Å)	5.79007(19)	21.6822(7)	16.5601(5)
β (°)	104.748(3)	96.876(3)	90
Volume (Å^3)	675.16(3)	746.79(4)	2577.73(11)
Z (molecules/cell)	4	4	8
Density calculated (Mg m^{-3})	1.501	1.663	1.383
Crystal size (mm)	0.22 x 0.18 x 0.06	0.26 x 0.18 x 0.06	0.60x0.22x0.18
Wavelength	0.71073	0.71073	0.71073
Absorption coefficient	0.474	0.792	0.097
F(000)	312	376	1120
θ range for data collection (°)	2.390 to 25.998	1.892 to 25.991	2.292 to 25.995
Reflections collected	2351	2494	5509
Independent reflections (all)	1329	1471	2532
Independent reflections [I > 2σ (I)]	1078	1212	2051
Data / restraints / parameters	1329 / 0 / 91	1471 / 0 / 100	2532/0/185
Goodness-of-fit on F^2	1.063	1.053	1.038
R values [$I > 2\sigma(I)$]	R_1 = 0.0416, wR_2 = 0.1037	R_1 = 0.0339, wR_2 = 0.0838	R_1 = 0.0405, wR_2 = 0.1030
R values [all data]	R_1 = 0.0532, wR_2 = 0.1110	R_1 = 0.0444, wR_2 = 0.0894	R_1=0.0528 wR_2 = 0.1106
$\Delta\rho_{max}$ and $\Delta\rho_{min}$ (eÅ^{-3})	0.182, -0.322	0.211, -0.240	0.142, -0.172

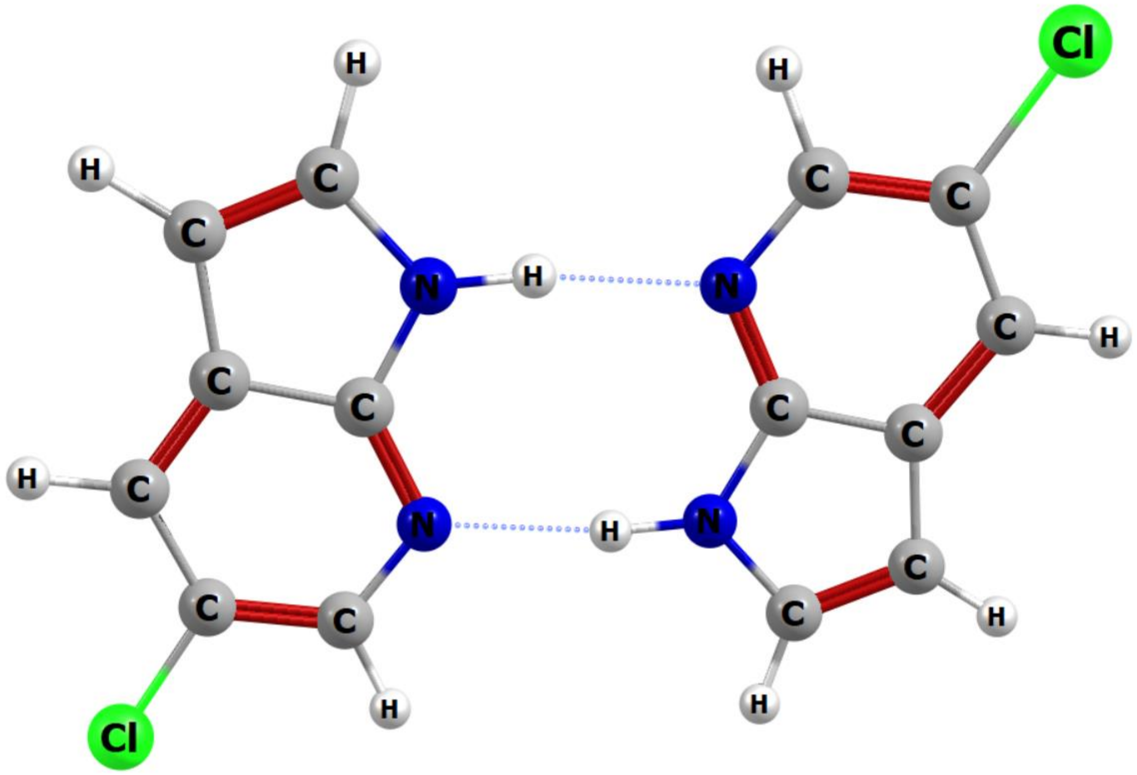

Scheme S1. Optimized geometry of 5Cl7AI dimer obtained at the ωB97X-D/6-31++G(d,p) level.

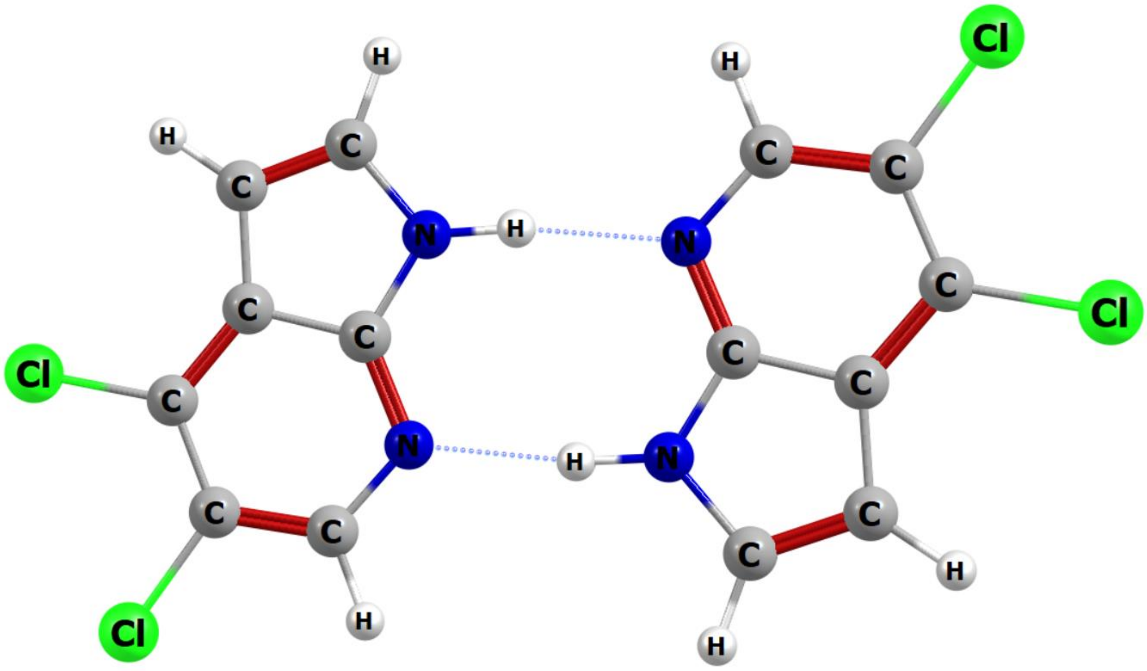

Scheme S2. Optimized geometry of 4,5Cl7AI dimer obtained at the ωB97X-D/6-31++G(d,p) level.

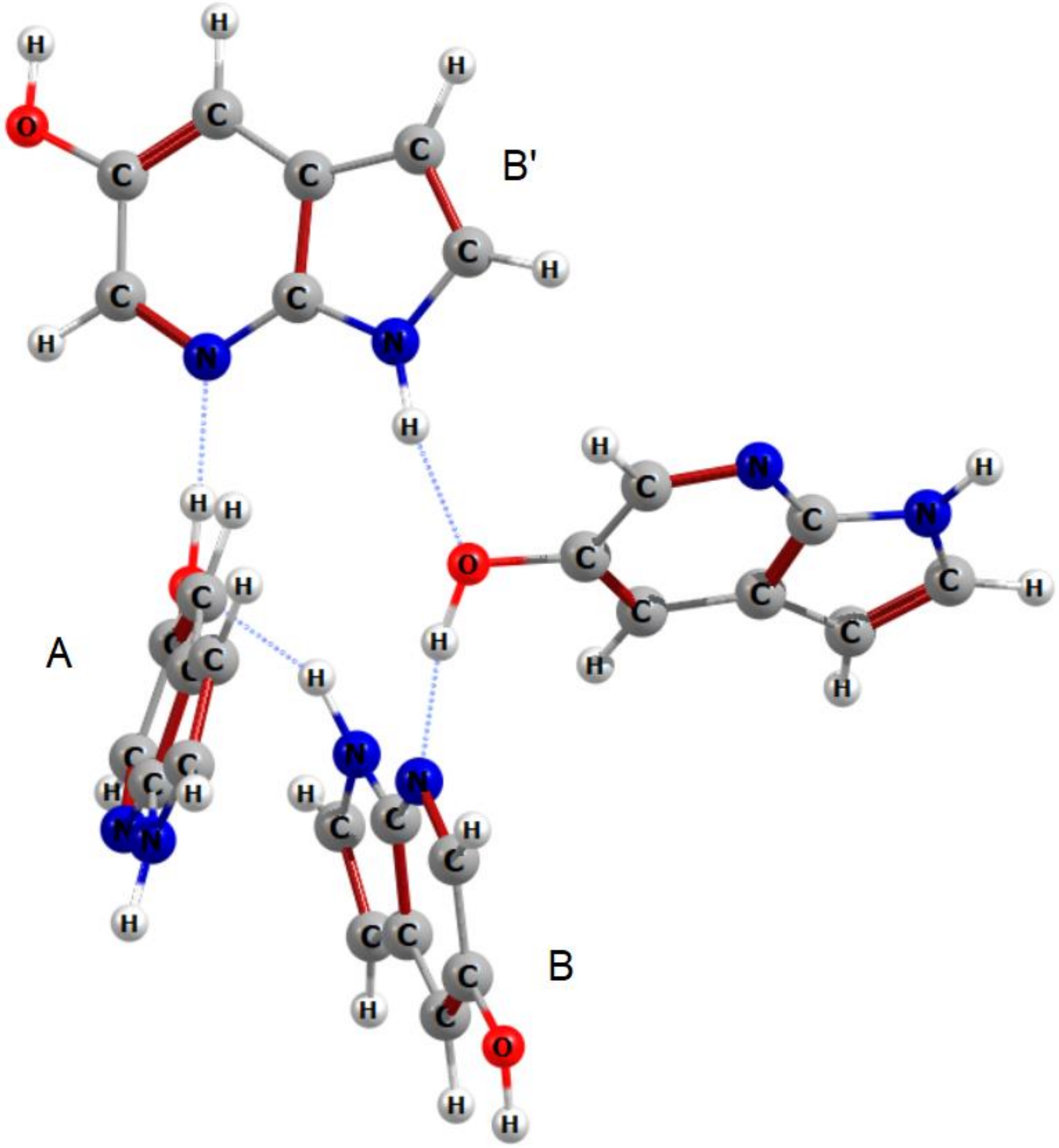

Scheme S3. Optimized geometry of 5OH7AI tetramer obtained at the B3LYP-D3/6-31++G(d,p) level.

Table S2. Selected experimental bond lengths and bond angles (X-ray) together with theoretical values calculated at the ωB97X-D/6-31++G(d,p) level for 5Cl7AIH and 4,5Cl7AIH, and at the B3LYP-D3/6-31++G(d,p) level for 5OH7AIH. Standard uncertainties (s.u.) are given in parentheses.

	**5Cl7AI**		**4,5Cl7AI**		**5OH7AI**			
	Exp.	Theor.	Exp.	Theor	Exp. A	Theor. A	Exp. B	Theor. B
N1–C2	1.367(3)	1.374	1.374(3)	1.374	1.361(2)	1.381	1.370(2)	1.382
C2–C3	1.354(3)	1.370	1.357(3)	1.370	1.345(3)	1.376	1.350(3)	1.376
C3–C3a	1.422(3)	1.432	1.415(3)	1.429	1.427(2)	1.435	1.425(2)	1.434
C3a–C4	1.383(3)	1.396	1.388(3)	1.364	1.388(2)	1.403	1.388(2)	1.402
C4–C5	1.377(3)	1.386	1.379(3)	1.392	1.379(2)	1.394	1.380(2)	1.393
C5–C6	1.389(3)	1.403	1.395(3)	1.405	1.392(2)	1.413	1.387(2)	1.409
C6–N7	1.331(2)	1.331	1.331(3)	1.328	1.334(2)	1.337	1.336(2)	1.337
N7–C7a	1.335(2)	1.335	1.332(3)	1.335	1.330(2)	1.331	1.3287(19)	1.334
N1–C7a	1.358(2)	1.365	1.360(2)	1.364	1.367(2)	1.380	1.363(2)	1.377
C3a–C7a	1.418(3)	1.420	1.417(3)	1.419	1.408(2)	1.425	1.410(2)	1.423
C4–Cl4			1.7217(19)	1.728				
C5–Cl5/O5	1.744(2)	1.747	1.733(2)	1.737	1.3658(18)	1.374	1.3680(19)	1.374
C2–N1–C7a	108.29(17)	108.2	108.65(16)	108.3	108.03(14)	108.9	108.16(14)	107.9
N1–C2–C3	110.65(18)	110.7	110.0(2)	110.7	110.73(15)	109.9	110.17(16)	110.5
C2–C3–C3a	106.79(18)	106.2	106.94(18)	106.0	107.16(16)	106.9	107.41(16)	106.6
C3–C3a–C7a	106.14(18)	106.5	106.84(17)	106.9	105.76(14)	106.8	105.76(14)	106.4
C4–C3a–C7a	117.15(17)	117.2	116.33(18)	116.9	117.45(14)	117.1	117.13(14)	117.1
C3a–C4–C5	117.18(18)	117.0	118.02(17)	117.8	117.94(14)	117.1	118.00(14)	117.7
C4–C5–C6	121.30(19)	121.2	120.49(18)	119.9	119.79(14)	120.2	119.95(14)	120.4
C5–C6–N7	123.43(18)	122.9	123.62(19)	123.9	123.89(15)	124.1	123.88(14)	122.9
C6–N7–C7a	114.92(16)	115.8	114.87(16)	115.5	115.51(13)	114.8	115.39(13)	116.6
C3a–C7a–N7	126.01(17)	125.9	126,66(17)	126.1	125.39(14)	126.7	125.65(14)	125.4
N1–C7a–C3a	108.13(16)	108.3	107.56(17)	108.0	108.32(14)	107.6	108.48(14)	108.5
N7–C7a–N1	125.86(17)	125.8	125.78(17)	125.9	126.27(14)	125.7	125.87(14)	126.2
C4–C3a–C3	136.67(19)	136.3	136.83(18)	136.2	136.72(16)	136.1	137.11(15)	136.4
Cl4–C4–C3a			119.81(16)	119.9				
Cl4–C4–C5			122.17(15)	122.3				
Cl5/O5–C5–C4	120.18(16)	120.2	121.95(15)	122.0	123.71(14)	122.9	123.55(14)	123.6
Cl5/O5–C5–C6	118.52(16)	118.5	117.55(16)	118.2	116.46(14)	116.8	116.50(14)	116.0

Table S3. Geometrical parameters of intermolecular interactions (Å, °) in the crystal structure of 5Cl7AI as determined by X-ray diffraction, and the corresponding theoretical values calculated at the ωB97X-D/6-31++G(d,p) level. Standard uncertainties (s.u.) for the experimental data are given in parentheses.

**D–H···A**	***d*** **(D–H)**	***d*** **(H···A)**	***d*** **(D···A)**	**D–H···A**
N1–H1···N7[i] (exp.)	0.86	2.16	2.983(2)	159.6
N1–H1···N7 (th.)	1.03	1.91	2.932	171.6
**C–H···π**	***d*** **(D–H)**	***d*** **(H···A)**	***d*** **(D···A)**	**D–H···A**
C4–H4···*Cg*1[ii] (exp.)	0.93	2.778	3.48952(8)	134.1
**π–π**	***Cg*2···*Cg*2[iii]**	**Interplanar distance**	**Centroid slippage**	
	3.73354(8)	3.41838(8)	1.50132(8)	

Symmetry codes: (i) *1 − x, 1 − y, 2 − z*; (ii) *x, 3/2 − y, −1/2 + z;* (iii) *1-x, 1-y, 1-z*. *Cg*1 and *Cg*2 are the centroids of the pyridine ring and 7-azaindole ring, respectively.

Table S4. Geometrical parameters of intermolecular interactions (Å, °) in the crystal structure of 4,5Cl7AI as determined by X-ray diffraction, and the corresponding theoretical values calculated at the ωB97X-D/6-31++G(d,p) level. Standard uncertainties (s.u.) for the experimental data are given in parentheses.

**D–H···A**	***d*** **(D–H)**	***d*** **(H···A)**	***d*** **(D···A)**	**D–H···A**
N1–H1···N7[i] (exp.)	0.86	2.09	2.939(2)	168.7
N1–H1···N7 (th.)	1.03	1.91	2.932	171.6
C2–H2···Cl2[ii](exp.)	0.93	2.92	3.730(2)	145.8
C3–H3···C3[iii](exp.)	0.93	2.88	3.798(4)	167.6

Symmetry codes: (i) *1 – x, –y, –z*; (ii) *1 + x, 1 + y, z*; (iii) *1 – x, 1/2 + y, 1/2 – z.*

Table S5. Geometrical parameters of intermolecular interactions (Å, °) in the crystal structure of 5OH7AI as determined by X-ray diffraction, and the corresponding theoretical values calculated at the B3LYP-D3/6-31++G(d,p) level. Standard uncertainties (s.u.) for the experimental data are given in parentheses.

**D–H···A**	**d (D–H)**	**d (H···A)**	**d (D···A)**	**D–H···A**
N1A–H1A···O1B[i](exp.)	0.86	2.15	2.938(2)	152
N1B–H1B···O1A(exp.)	0.86	2.23	2.941(2)	141
N1B–H1B···O1A(th.)	1.02	1.97	2.898	149
N1B–H1B···O1A(th.)	1.02	1.84	2.848	166
O1A–H11A···N7B[ii](exp.)	0.93(2)	1.73(2)	2.643(2)	166(2)
O1A–H11A···N7B(th.)	1.00	1.69	2.685	168
O1A–H11A···N7B(th.)	1.00	1.71	2.709	175
O1B–H11B···N7A[iii](exp.)	0.88(3)	1.79(3)	2.663(2)	172(2)
C2A–H2A···O1A[iv](exp.)	0.93	2.42	3.250(2)	148

Symmetry codes: (i) *x, 2 – y, –½ + z*; (ii) *1 – x, 1 – y, 2 – z*; (iii) *3/2 – x, 3/2 – y, 1/2 + z*; *(iv) x, 1 + y, z*.

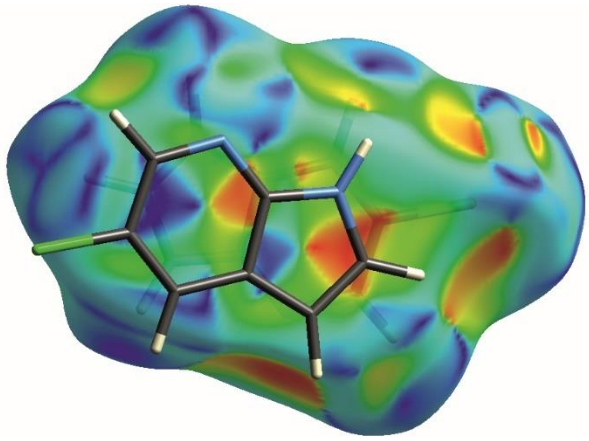

Figure S1. Hirshfeld surface of 5Cl7AI mapped over the shape index, showing complementary red and blue triangular regions that indicate π···π stacking interactions between aromatic rings.

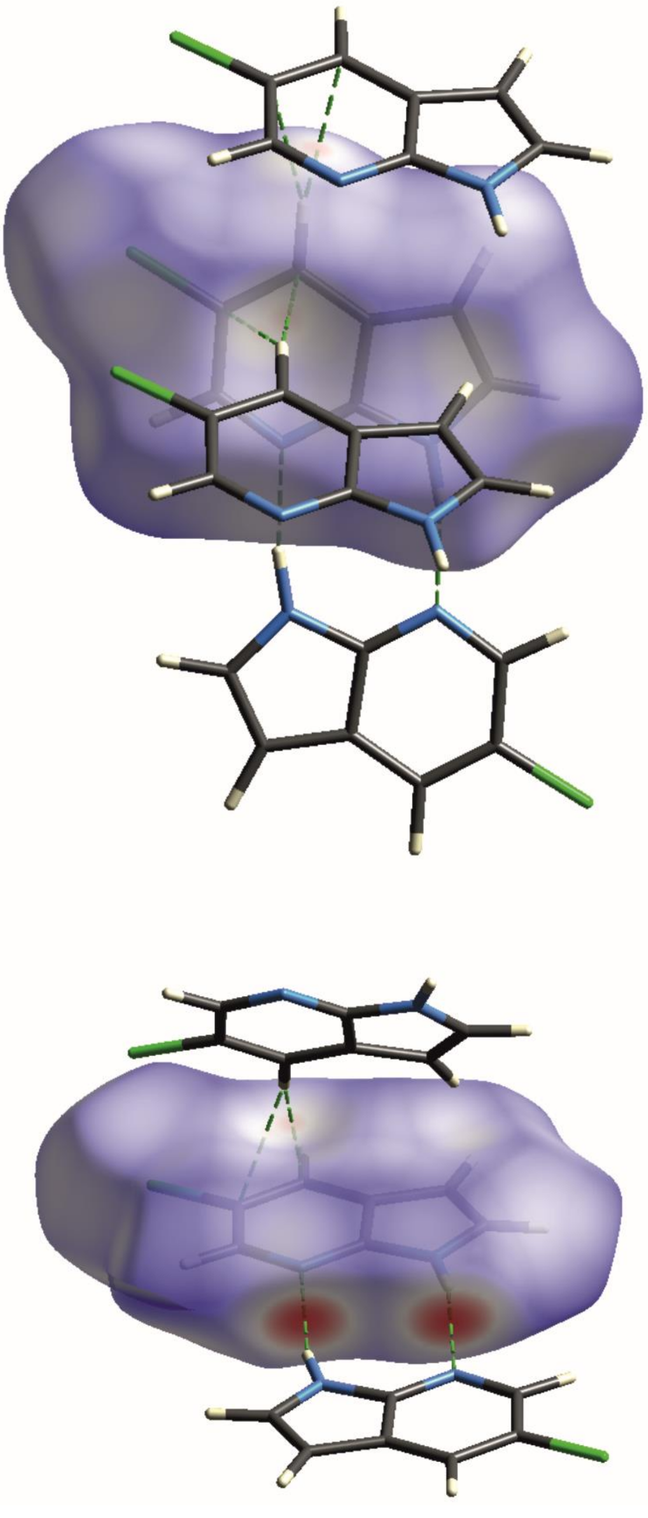

Figure S2. Red regions on the Hirshfeld surface of 5Cl7AI mapped over d_{norm} indicate N–H···N hydrogen bonds and H···H/H···Cl contacts stabilizing the crystal packing.

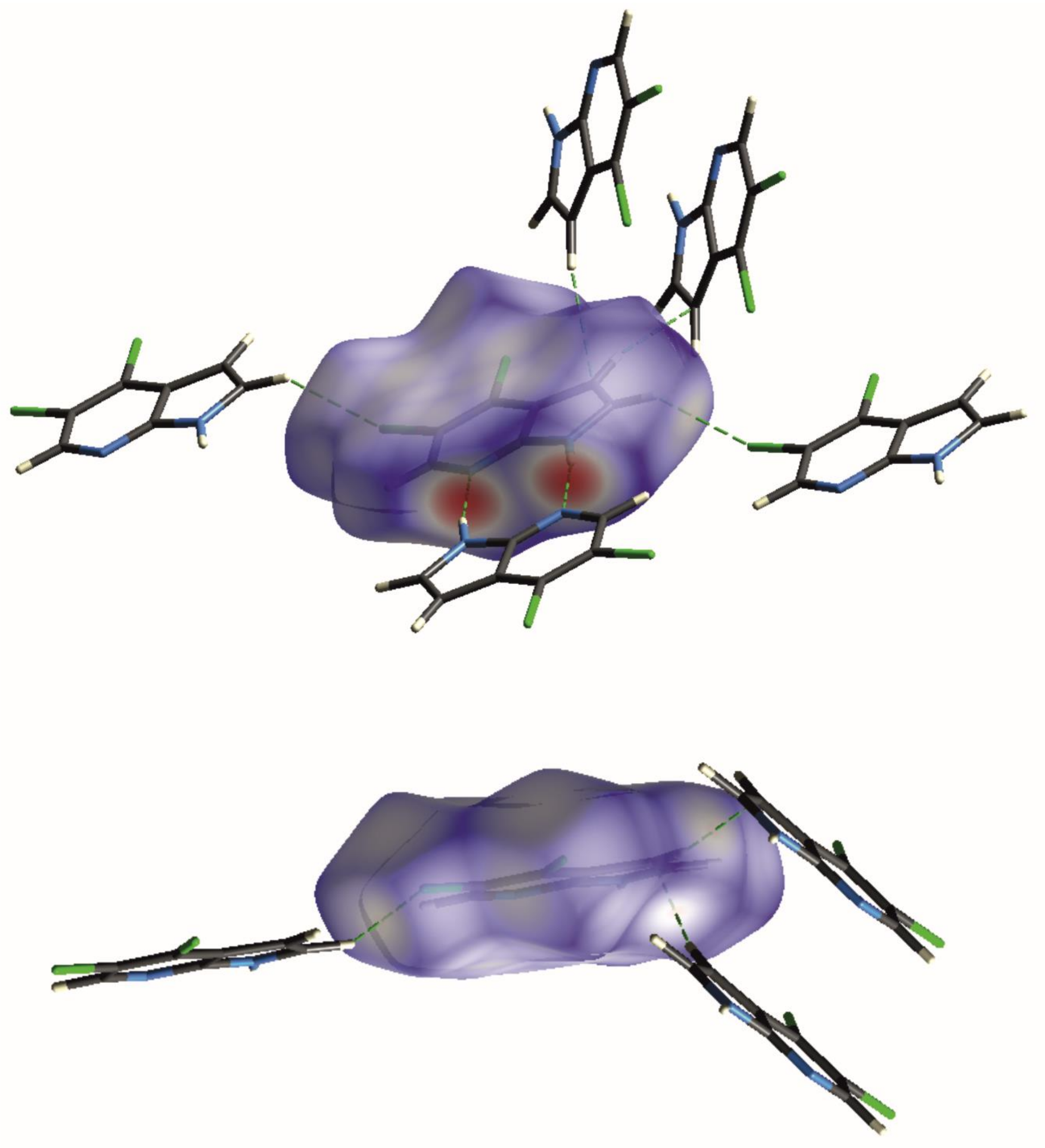

Figure S3. Red regions on the Hirshfeld surface of 4,5Cl7AI mapped over d_{norm} indicate N–H···N hydrogen bonds and weaker C–H···Cl and C–H···C contacts stabilizing the crystal packing.

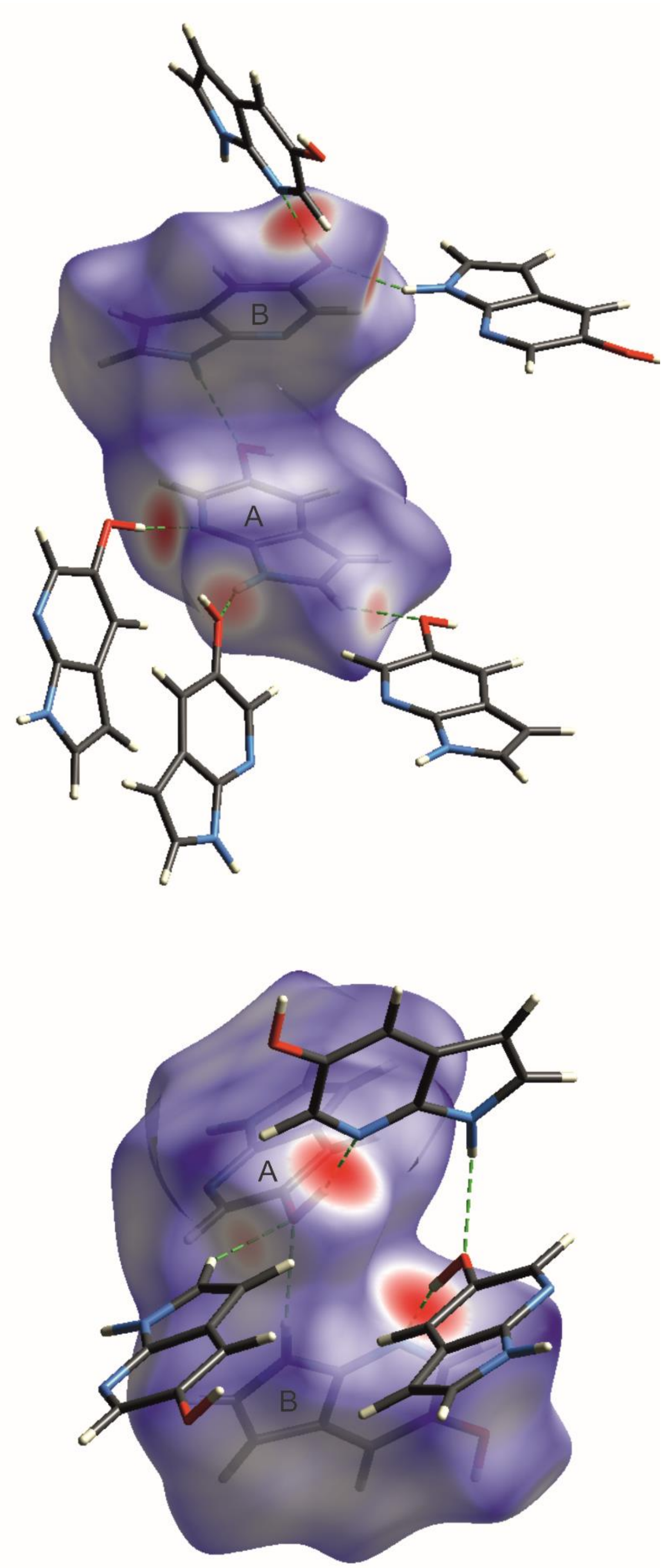

Figure S4. Red regions on the Hirshfeld surfaces of 5OH7AI (molecules A and B) mapped over d_{norm} indicate N–H···O, O–H···N, and weak C–H···O hydrogen bonds stabilizing the packing.

Table S6. Experimental (FT-IR and FT-Raman) and calculated wavenumbers (ṽ, cm^{-1}), infrared intensities (A^{IR}, $km \cdot mol^{-1}$), and Raman scattering activities (S^{R}, $Å^{4} \cdot amu^{-1}$) for 5Cl7AIH obtained at the ωB97X-D/6-31++G(d,p) level.

FT-IR	FT-Raman	$\tilde{\nu}^{a}$	Sym.	A^{IR}	S^{R}	Mode description[c]
3300 – 2500		3152 (A_u)	A_u	3727	0	$\nu_{as}(NH_{HB})$
		3116 (A_g)	A_g	0	1107	$\nu_{s}(NH_{HB})$
3131	3131	3128[b], 3129	A_u, A_g	5	416	ν(C3H), ν(C2H)
	3112	3111, 3111	A_u, A_g	1	134	ν(C2H), ν(C3H)
	3069	3067[b], 3067	A_u, A_g	6	223	ν(C4H)
3052	3056	3055[b], 3055	A_u, A_g	20	125	ν(C6H)
1599	1596	1614, 1606	A_u, A_g	5	13	ν(R6)
1573	1579	1583[b], 1589	A_u, A_g	67	7	ν(R6)
1495	1496	1497[b], 1499	A_u, A_g	48	230	ν(R5), $\delta(NH_{HB})$
1469		1481[b], 1489	A_u, A_g	90	32	$\delta(NH_{HB})$, ν(R5), ν(R6)
1434	1444	1444[b], 1449	A_u, A_g	44	9	$\delta(NH_{HB})$, ν(R5), δ(CH)
1402	1411	1399[b], 1407	A_u, A_g	133	52	ν(R5)
1336	1342	1325[b], 1326	A_u, A_g	81	28	δ(CH)
1301	1301	1299, 1293	A_u, A_g	3	46	$\delta(NH_{HB})$, δ(CH), ν(R6)
1283	1285	1285[b],1288	A_u, A_g	117	59	ν(R6)
1245	1246	1225, 1224	A_u, A_g	13	10	δ(CH)
1189	1190	1184, 1183	A_u, A_g	30	15	ν(R5)
1109	1113	1112[b], 1114	A_u, A_g	57	14	ν(R5), δ(CH)
1082	1087	1072[b], 1073	A_u, A_g	28	30	δ(CH), ν(R6)
1067	1070	1054, 1054	A_u, A_g	20	55	δ(CH), ν(R5)
934	933	932, 931	A_u, A_g	95	17	δ(R6), ν(C5Cl)
906	908	915, 915	A_u, A_g	10	0.01	γ(CH)
890	890	894[b], 895	A_u, A_g	48	1	γ(CH)
877	869	890[b], 885[b]	A_u, A_g	61	57	δ(R5), ν(R5/R6)
		886[b], 882	A_u, A_g	1	1	γ(CH)
863		841 (A_u)	A_u	149	0	$\gamma_{as}(NH_{HB})$
		823 (A_g)	A_g	0	2	$\gamma_{s}(NH_{HB})$
785	789	783[b], 784	A_u, A_g	46	36	ν(R5)
	764	780, 774	A_u, A_g	1	1	τ(R5/R6)
733	744	721[b], 723	A_u, A_g	89	1	γ(CH)
690	683	698, 687	A_u, A_g	56	7	ν(C5Cl), δ(R6)
	611	608[b], 607	A_u, A_g	4	10	δ(R5/R6)
604	605	610[b], 607[b]	A_u, A_g	13	1	τ(R5/R6)
572	576	576[b], 578	A_u, A_g	0.4	1	γ(C5Cl), τ(R5)
457	452	461, 452	A_u, A_g	20	1	δ(R5/R6), δ(R6)
434	438	434[b], 436	A_u, A_g	4	1	γ(C5Cl), τ(R5/R6), +γ(C4H)
378	380	375[b], 375	A_u, A_g	6	21	ν(C5Cl), δ(R6)
333	334	325, 322	A_u, A_g	5	0.4	τ(R5/R6)
254	263	244, 243	A_u, A_g	9	3	δ(C5Cl)
244	242	241, 239	A_u, A_g	4	0.1	τ(R5/R6)
155	144	129, 127	A_u, A_g	0.3	4	τ(R6), +γ(C5Cl)
	144	107 (A_g)	A_g	0	3	$dimer_{strech, ip bend}$
		77 (A_g)	A_g	0	6	$dimer_{oop, wag}$
		68 (A_g)	A_g	0	2	$dimer_{ip bend}$
70		54 (A_u)	A_u	8	0	$dimer_{strech, ip bend}$
		28 (A_u)	A_u	1	0	$dimer_{oop wag}$
		18 (A_u)	A_u	0.5	0	$dimer_{oop twist}$

Abbreviations: ov, overlapped; ν, stretching; δ, in-plane bending; γ, out-of-plane bending; τ, torsion; 5R, five-membered pyrrole ring; 6R, six-membered pyridine ring.

Subscripts: HB, hydrogen atom involved in hydrogen bonding; as, antisymmetric; s, symmetric with respect to the center of symmetry in the dimer; stretch, stretching; ip, in-plane; opp, out-of-plane; bend, bending; wag, wagging; twist, twisting.
Remark: For normal modes of *Au* symmetry, the theoretical Raman scattering activity (S^R) is equal to zero, whereas for *Ag* modes, the predicted infrared intensity (A^{IR}) is equal to zero.
[a] Calculated frequencies were scaled. Details are given in Section 3.4.
[b] Theoretical frequencies were reordered.
[c] Assignments obtained from PED analysis calculated by FCART07 and verified using the Chemcraft program.

Table S7. Experimental (FT-IR and FT-Raman) and calculated wavenumbers (ṽ, cm⁻¹), infrared intensities (A^{IR}, km·mol⁻¹), and Raman scattering activities (S^R, Å⁴·amu⁻¹) for 4,5Cl7AIH obtained at the ωB97X-D/6-31++G(d,p) level.

FT-IR	FT-Raman	$\tilde{\nu}^a$	Sym.	A^{IR}	S^R	Mode description[c]
3300 – 2500		3153 (A_u)	A_u	4118	0	$\nu_{as}(NH_{HB})$
		3118 (A_g)	A_g	0	538	$\nu_s(NH_{HB})$
	3142	3136, 3136	A_u, A_g	4	145	ν(C3H), ν(C2H)
3111	3127	3117, 3116	A_u, A_g	3	131	ν(C2H), ν(C3H)
	3055	3057, 3057	A_u, A_g	17	60	ν(C6H)
1596		1605, 1596	A_u, A_g	24	11	ν(R6)
1566	1577	1572[b], 1581	A_u, A_g	243	44	ν(R6)
1490	1492	1496[b], 1495	A_u, A_g	68	542	ν(R5/R6), δ(CH)
1462	1475	1470[b], 1481	A_u, A_g	77	92	ν(R6), $\delta(NH_{HB})$
1445	1448	1447[b], 1448	A_u, A_g	47	25	δ(CH), ν(R6), $\delta(NH_{HB})$
1398	1411	1391[b], 1402	A_u, A_g	91	121	ν(R5)
1336	1342	1327[b], 1331	A_u, A_g	173	89	δ(CH)
1293	1299	1289[b], 1290	A_u, A_g	136	494	ν(R6)
1285	1277	1285, 1277	A_u, A_g	30	198	δ(CH), $\delta(NH_{HB})$, ν(R6)
1199	1194	1188, 1186	A_u, A_g	30	56	ν(R5)
1181	1185	1177, 1175	A_u, A_g	101	63	ν(R6), δ(CH)
1116	1120	1109[b], 1115	A_u, A_g	7	37	ν(R5), δ(CH)
1082	1071	1065, 1064	A_u, A_g	12	315	δ(CH), ν(R5)
968	969	966, 966	A_u, A_g	45	142	ν(C4Cl), δ(R6)
923	920	924, 923	A_u, A_g	17	158	δ(R5), ν(R5/R6)
	894	899[b], 900	A_u, A_g	11	2	γ(CH)
884	868	891, 887	A_u, A_g	15	12	γ(CH)
852	852	847, 844	A_u, A_g	218	30	δ(R5)
ov		840	A_u	140	0	$\gamma_{as}(NH_{HB})$
	ov	821	A_g	0	13	$\gamma_s(NH_{HB})$
783	794	783, 779	A_u, A_g	12	2	γ(CH), τ(R5/R6)
765	716	723[b], 724	A_u, A_g	78	5	γ(CH)
709	703	711, 700	A_u, A_g	82	16	ν(C5Cl), δ(R6)
650	653	651, 649	A_u, A_g	0.2	178	δ(R5/R6), ν(C4Cl)
611	612	621, 620	A_u, A_g	4	3	γ(C4Cl), γ(C5Cl)
594	596	603, 599	A_u, A_g	14	6	τ(R5)
531	528	533, 522[b]	A_u, A_g	21	111	δ(R5/R6), δ(R5/R6), δ(C4Cl)
523	520	531, 527	A_u, A_g	0.3	14	τ(R5/R6), τ(R6), γ(C4Cl)
442	452	442[b], 449	A_u, A_g	2	508	ν(C4Cl), δ(R6)
370	368	364, 361	A_u, A_g	8	215	ν(C5Cl), δ(R6)
339	336	338, 333	A_u, A_g	1	12	τ(R6), γ(C5Cl)
277	277	254[b], 254	A_u, A_g	7	2	τ(R5/R6)
240	238	241, 240	A_u, A_g	16	88	δ(C5Cl), δ(C4Cl)
192	191	192[b], 193	A_u, A_g	0.3	134	δ(C4Cl)
	177	165[b], 173	A_u, A_g	0.01	101	τ(R5/R6)
120	117	99, 97	A_u, A_g	1	118	τ(R5/R6)
	107	95 (A_g)	A_g	0	148	$\text{dimer}_{\text{stretch, ip bend}}$
		67 (A_g)	A_g	0	107	$\text{dimer}_{\text{ip bend,}}$
		58 (A_g)	A_g	0	621	$\text{dimer}_{\text{oop wag}}$
57		48 (A_u)	A_u	6	0	$\text{dimer}_{\text{stretch, ip bend}}$
		19 (A_u)	A_u	0.1	0	$\text{dimer}_{\text{oop wag}}$
		17 (A_u)	A_u	0.3	0	$\text{dimer}_{\text{oop twist}}$

Abbreviations and notations are the same as in Table S6.

Table S8. Experimental (FT-IR and FT-Raman) and calculated wavenumbers ($\tilde{\nu}$, cm^{-1}), infrared intensities (A^{IR}, $km \cdot mol^{-1}$), and Raman scattering activities (S^{R}, $Å^{4} \cdot amu^{-1}$) for 5OH7AIH obtained at the B3LYP-D3/6-31++G(d,p) level.

FT-IR	FT-Raman	$\tilde{\nu}$[a]	A^{IR}	S^{R}	Mode description[b]
		3684	44	264	$\nu(OH_F)_{B'}$
		3684	90	98	$\nu(OH_F)_{B}$
		3538	79	145	$\nu(NH_F)_{A'}$
		3537	96	173	$\nu(NH_F)_{A}$
3315		3321	464	128	$\nu(NH_{HB})_{B}$
		3252	1290	341	$\nu(NH_{HB})_{B'}$
		3149	1	168	$\nu(C2H)_{B'}$, $\nu(C3H)_{B'}$
		3145	1	183	$\nu(C2H)_{B}$, $\nu(C3H)_{B}$
		3144	2	189	$\nu(C2H)_{A}$, $\nu(C3H)_{A}$
		3143	2	170	$\nu(C2H)_{A'}$, $\nu(C3H)_{A'}$
3131	3132	3129	2	114	$\nu(C3H)_{B'}$, $\nu(C2H)_{B'}$
		3127	2	103	$\nu(C3H)_{B}$, $\nu(C2H)_{B}$
		3125	2	66	$\nu(C3H)_{A}$, $\nu(C2H)_{A}$
		3125	2	74	$\nu(C3H)_{A'}$, $\nu(C2H)_{A'}$
		3089	11	130	$\nu(C4H)_{A'}$
		3082	4	84	$\nu(C4H)_{A}$
3104	3109	3080	3	49	$\nu(C6H)_{B}$
		3079	7	106	$\nu(C6H)_{B'}$
		3058	9	64	$\nu(C6H)_{A'}$
		3053	16	93	$\nu(C6H)_{A}$
3036	3040	3047	13	153	$\nu(C4H)_{B}$
		3047	17	175	$\nu(C4H)_{B'}$
2800 – 2000		2939	3591	512	$\nu(OH_{HB})_{A'}$
		2854	1818	505	$\nu(OH_{HB})_{A}$
		1627	28	5	$\nu(R6)_{B'}$
		1626	10	7	$\nu(R6)_{B}$
		1622	25	7	$\nu(R6)_{A'}$
		1619	18	9	$\nu(R6)_{A}$
1594	1594	1606	44	1	$\nu(R6)_{B}$
		1605	16	5	$\nu(R6)_{B'}$
		1595	8	3	$\nu(R6)_{A'}$
		1591	6	2	$\nu(R6)_{A}$
		1516	93	85	$\delta(NH_{HB})_{B',B}$, $\nu(R6)_{B'}$
		1513	67	13	$\nu(R5/R6)_{A'}$, $\delta(CH)_{A'}$
		1511	27	162	$\nu(R5/R6)_{B}$, $\delta(CH)_{B}$
		1510	19	117	$\nu(R5/R6)_{A}$, $\delta(CH)_{A}$
		1506	25	40	$\nu(R5)_{A',B'}$, $\delta(CH)_{A',B'}$
1494	1504	1505	62	47	$\nu(R6)_{B}$, $\delta(NH_{HB})_{B}$, $\delta(CH)_{B}$
		1502	126	72	$\nu(R5)_{A',B'}$, $\delta(CH)_{A'}$, $\delta(OH_{HB})_{A'}$
		1498	82	31	$\nu(R5/R6)_{A}$, $\delta(OH_{HB})_{A}$, $\delta(NH_F)_{A}$
1469		1472	27	4	$\nu(R5)_{B'}$, $\delta(NH_{HB})_{B'}$, $\delta(CH)_{B'}$
		1462	28	5	$\delta(OH_{HB})_{A'}$, $\delta(OH_F)_{B'}$
		1456	68	3	$\delta(NH_{HB})_{B}$, $\delta(OH_{HB})_{A}$
		1453	33	5	$\delta(OH_{HB})_{A}$, $\delta(NH_{HB})_{B}$
1428	1431	1433	63	10	$\nu(R5/R6)_{B'}$
		1433	3	22	$\nu(R5/R6)_{B}$, $\delta(CH)_{B}$
		1417	20	50	$\nu(R5/R6)_{A}$
		1416	12	70	$\nu(R5/R6)_{A'}$
1394		1384	36	15	$\nu(R6)_{A}$, $\delta(OH_{HB})_{A}$
		1381	97	9	$\nu(R6)_{A'}$, $\delta(OH_{HB})_{A'}$
		1362	12	18	$\delta(OH_F)_{B'}$, $\nu(R6)_{B'}$
		1355	27	13	$\nu(R6)_{B}$, $\delta(OH_F)_{B}$

FT-IR	FT-Raman	$\tilde{\nu}$[a]	A^{IR}	S^{R}	Mode description[b]
		1341	6	23	δ(CH)$_{B}$, ν(R5)$_{B}$
1344	1347	1340	86	18	δ(CH)$_{B'}$, ν(R5)$_{B'}$
		1340	21	17	δ(CH)$_{A'}$, ν(R6)$_{A'}$
		1337	37	19	δ(CH)$_{A}$, ν(R6)$_{A}$
		1320	20	42	ν(R5/R6)$_{B}$, ν(C-O$_{F}$)$_{B}$
		1319	22	17	ν(R5/R6)$_{B'}$, ν(C-O$_{F}$)$_{B'}$
		1317	64	37	ν(R6)$_{A'}$
1299	1299	1313	10	84	ν(R6)$_{A}$, ν(C-O$_{HB}$)$_{A}$
		1273	10	16	δ(CH)$_{B'}$, ν(C-O$_{F}$)$_{B'}$
		1269	38	4	δ(CH)$_{B}$, ν(C-O$_{F}$)$_{B}$
1277	1277	1267	131	13	ν(R6)$_{A}$, δ(CH)$_{A}$
		1263	43	20	ν(R6)$_{A'}$, δ(CH)$_{A'}$
1252	1254	1249	92	3	δ(CH)$_{A'}$, ν(C-O$_{HB}$)$_{A'}$
		1248	68	6	δ(CH)$_{A}$, ν(C-O$_{HB}$)$_{A}$
		1227	69	3	δ(CH)$_{B}$
	1227	1225	17	13	δ(CH)$_{B'}$
		1222	49	12	δ(CH)$_{A'}$
1212	1215	1220	39	5	δ(CH)$_{A}$, ν(C-O$_{HB}$)$_{A}$, ν(R6)$_{A}$
		1162	241	10	ν(R5)$_{B'}$, ν(C-O$_{F}$)$_{B'}$, δ(OH$_{F}$)$_{B'}$
		1160	175	8	ν(C-O$_{F}$)$_{B}$, ν(R5)$_{B}$, δ(OH$_{F}$)$_{B}$
		1152	93	5	δ(OH$_{F}$)$_{B}$, δ(CH)$_{B}$, ν(R6)$_{B}$
		1151	128	4	δ(OH$_{F}$)$_{B'}$, δ(CH)$_{B'}$
		1150	50	9	ν(R5)$_{A}$, δ(OH$_{F}$)$_{B'}$, δ(CH)$_{A}$, ν(C-O$_{HB}$)$_{A}$
1149	1152	1150	64	7	ν(R5)$_{A'}$, ν(C-O$_{HB}$)$_{A'}$, δ(CH)$_{A'}$
		1124	31	1	δ(CH)$_{B'}$, ν(R5)$_{B'}$, δ(NH$_{HB}$)$_{B'}$
1099	1101	1115	21	1	δ(CH)$_{B}$, ν(R5)$_{B}$, δ(NH$_{HB}$)$_{B}$
		1090	22	3	δ(CH)$_{A}$, ν(R5)$_{A}$, δ(NH$_{F}$)$_{A}$
		1089	17	4	δ(CH)$_{A'}$, ν(R5)$_{A'}$, δ(NH$_{F}$)$_{A'}$
		1073	3	58	δ(CH)$_{B'}$, ν(R5)$_{B'}$
		1071	6	11	δ(CH)$_{B}$, ν(R5)$_{B}$
		1066	13	33	δ(CH)$_{A}$, ν(R5)$_{A}$
1072	1075	1065	13	26	δ(CH)$_{A'}$, ν(R5)$_{A'}$
ov		1005	25	8	γ(OH$_{HB}$)$_{A'}$
ov		998	11	3	γ(OH$_{HB}$)$_{A}$
		998	5	21	ν(R6)$_{A}$, δ(R6)$_{A}$, δ(CH)$_{A}$
980	985	997	10	4	δ(R6)$_{A'}$, ν(R6)$_{A'}$, δ(CH)$_{A'}$
ov		979	27	33	γ(OH$_{HB}$)$_{A'}$
ov		967	40	1	γ(OH$_{HB}$)$_{A}$
		922	4	1	γ(CH)$_{A}$
		917	18	6	δ(R5)$_{B}$, ν(R6)$_{B}$
		913	5	1	γ(CH)$_{A'}$
891	886	906	27	14	δ(R5)$_{B'}$, ν(R6)$_{B'}$, δ(CH)$_{B'}$
		905	13	12	δ(R5)$_{A'}$, ν(R6)$_{A'}$, δ(CH)$_{A'}$
		904	24	14	δ(R5)$_{A}$, ν(R6)$_{A}$, δ(CH)$_{A}$
		902	16	1	γ(CH)$_{B}$
		901	2	1	γ(CH)$_{B'}$
		900	25	2	γ(CH)$_{A}$
		893	24	1	γ(CH)$_{A'}$
		889	13	2	γ(CH)$_{B',A'}$
		883	23	1	γ(CH)$_{B}$
		873	28	0	γ(CH)$_{B'}$
865	858	870	11	2	γ(CH)$_{B}$
		864	1	3	γ(CH)$_{A}$
		859	0	2	γ(CH)$_{A'}$
ov		821	3	8	γ(NH$_{HB}$)$_{B'}$, δ(R5/R6)$_{B'}$
801	805	820	25	0	ν(R5/R6)$_{B}$, δ(R5/R6)$_{B}$

FT-IR	FT-Raman	$\tilde{\nu}$[a]	A^{IR}	S^{R}	Mode description[b]
		818	2	22	$\nu(R5/R7)_{A'}$, $\delta(R5/R6)_{A'}$
		818	29	21	$\nu(R5/R6)_{A}$, $\delta(R5/R6)_{A}$
ov		794	40	7	$\gamma(NH_{HB})_{B'}$, $\gamma(CH)_{B'}$, $\gamma(R5/R6)_{B'}$
		793	26	15	$\nu(R6)_{B}$, $\delta(R6)_{B}$
		790	1	1	$\tau(R5/R6)_{A}$
		787	1	0	$\tau(R5/R6)_{A'}$
		783	16	9	$\tau(R5/R6)_{B}$, $\gamma(CH)_{B}$
774	778	782	37	23	$\nu(R6)_{B'}$, $\delta(R5/R6)_{B}$
ov		761	54	1	$\gamma(NH_{HB})_{B,B'}$, $\gamma(R5/R6)_{B'}$
		754	4	7	$\delta(R5/R6)_{A'}$, $\nu(R6)_{A'}$
		754	6	13	$\delta(R5/R6)_{A}$, $\nu(R6)_{A}$
719	724	734	78	1	$\gamma(CH)_{B}$
		731	44	1	$\gamma(CH)_{B'}$
		728	59	1	$\gamma(CH)_{A}$
		725	77	1	$\gamma(CH)_{A'}$
		699	48	3	$\gamma(OH_{HB})_{A}$, $\gamma(NH_{HB})_{B,}$
654		644	2	1	$\gamma(C\text{-}O_{HB})_{A}$, $\tau(R5/R6)_{A}$
		644	2	6	$\delta(C\text{-}O_{HB})_{A'}$, $\delta(R5/R6)_{A'}$
		644	1	3	$\tau(R5/R6)_{A'}$, $\gamma(C\text{-}O_{HB})_{A}$
		642	3	7	$\delta(C\text{-}O_{HB})_{A}$
		640	12	2	$\delta(C\text{-}O_{F})_{B'}$
	637	637	7	3	$\tau(R5/R6)_{A'}$
		627	8	1	$\gamma(C\text{-}O_{F})_{B'}$, $\tau(R5/R6)_{B'}$
		627	7	3	$\gamma(C\text{-}O_{F})_{B}$, $\tau(R5/R6)_{B}$
614	626	605	3	0	$\tau(R5/R6)_{A}$, $\gamma(C\text{-}O_{HB})_{A}$
546	551	602	2	0	$\tau(R5/R6)_{A'}$
		589	5	1	$\tau(R5/R6)_{B}$, $\gamma(C\text{-}O_{F})_{B}$
		583	3	0	$\tau(R5/R6)_{B'}$, $\gamma(C\text{-}O_{F})_{B'}$
		498	7	5	$\delta(C\text{-}O_{HB})_{A'}$, $\delta(R5/R6)_{A'}$
		494	7	3	$\delta(C\text{-}O_{HB})_{A}$, $\delta(R5/R6)_{A}$
494		491	3	12	$\delta(R6)_{A}$, $\delta(C\text{-}O_{HB})_{A}$
		488	4	3	$\delta(C\text{-}O_{HB})_{A'}$, $\delta(R5/R6)_{A'}$
484	487	487	2	14	$\delta(R6)_{A'}$
472		486	12	1	$\delta(C\text{-}O_{HB})_{A}$, $\delta(R5/R6)_{A}$
		477	17	3	$\delta(C\text{-}O_{F})_{B'}$, $\delta(R5)_{B'}$
		475	17	2	$\delta(C\text{-}O_{F})_{B}$, $\delta(R5/R6)_{B}$, $\gamma(NH_{F})_{A'}$
		466	1	2	$\gamma(C\text{-}O_{F})_{B}$, $\tau(R5/R6)_{B}$
		460	15	1	$\gamma(C\text{-}O_{F})_{B'}$, $\tau(R5/R6)_{B'}$
447	451	459	11	2	$\tau(R5/R6)_{A'}$
		457	1	1	$\gamma(C\text{-}O_{F})_{B'}$, $\tau(R6)_{B'}$
		428	48	2	$\gamma(NH_{F})_{A}$
		416	44	1	$\gamma(NH_{F})_{A'}$
373		380	20	0	$\tau(R5/R6)_{A}$, $\nu(OH_{A}\cdots N_{B})$
365	362	376	20	0	$\tau(R5/R6)_{A'}$, $\gamma(OH_{A'}\cdots N_{B'})$
		364	10	1	$\gamma(OH_{A'}\cdots N_{B'})$, $\tau(R5/R6)_{A'}$, $\delta(R5/R6)_{A'}$
		362	8	0	$\tau(R5/R6)_{B}$
		358	7	0	$\tau(R5/R6)_{B'}$
356		357	14	2	$\tau(R5/R6)_{A,B'}$, $\delta(C\text{-}O_{HB})_{A}$
		335	10	2	$\delta(C\text{-}O_{F})_{B'}$, $\delta(R5/R6)_{B'}$
		333	5	1	$\delta(C\text{-}O_{F})_{B}$, $\delta(R5/R6)_{B}$
		264	102	2	$\gamma(C\text{-}OH_{F})_{B}$
		252	98	2	$\gamma(C\text{-}OH_{F})_{B'}$
		252	4	0	$\tau(R5/R6)_{B}$, $\gamma(C\text{-}OH_{F})_{B'}$
256	260	244	3	0	$\tau(R5/R6)_{B'}$
250	250	241	8	0	$\tau(R5/R6)_{A,A'}$
		240	5	0	$\tau(R5/R6)_{A',A}$

FT-IR	FT-Raman	$\tilde{\nu}$[a]	A^{IR}	S^{R}	Mode description[b]
207	207	219	8	1	$\nu(NH_{B'}\cdots O_{A})$, $\nu(OH_{A}\cdots N_{B})$, $\tau(R5/R6)_{A}$
182	185	189	1	1	$\nu(NH_{B}\cdots O_{A'})$, $\tau(R5/R6)_{A'}$,$\nu(OH_{A'}\cdots N_{B'})$
	163	164	3	0	$\tau(R5/R6)_{B}$
		157	1	1	$\tau(R5/R6)_{B'}$
149	141	145	2	6	$\nu(NH_{B}\cdots O_{A'})$, $\tau(R5/R6)_{A'}$, $\gamma(OH_{A'}\cdots N_{B'})$
135		132	3	7	$\nu(OH_{A'}\cdots N_{B'})$, $\nu(OH_{A}\cdots N_{B})$, $\tau(R5/R6)_{A}$
120		111	6	1	$\nu(OH_{A'}\cdots N_{B'})$, $\nu(NH_{B}\cdots O_{A'})$, $\gamma(NH_{B}\cdots O_{A'})$, $\nu(OH_{A}\cdots N_{B})$
94	98	97	4	2	$\nu(NH_{B'}\cdots O_{A})$, $\nu(OH_{A}\cdots N_{B})$
86	84s	79	2	3	$\gamma(OH_{A'}\cdots N_{B'})$, $\gamma(NH_{B'}\cdots O_{A})$
77		73	3	3	$\delta(OH_{A}\cdots N_{B})$
61		64	3	1	$\gamma(OH_{A'}\cdots N_{B'})$, $\gamma(NH_{B}\cdots O_{A'})$, $\delta(NH_{B'}\cdots O_{A})$
		54	0	4	$\gamma(OH_{A}\cdots N_{B})$
		51	0	2	$\gamma(OH_{A'}\cdots N_{B'})$, $\gamma(NH_{B}\cdots O_{A'})$, $\delta(NH_{B'}\cdots O_{A})$
		49	1	2	$\gamma(OH_{A'}\cdots N_{B'})$
		39	1	3	$\gamma(NH_{B'}\cdots O_{A})$, $\gamma(OH_{A'}\cdots N_{B'})$
		30	1	2	$\gamma(OH_{A'}\cdots N_{B'})$
		26	0	4	$\gamma(NH_{B}\cdots O_{A'})$, $\gamma(OH_{A}\cdots N_{B})$, $\delta(OH_{A'}\cdots N_{B'})$
		20	1	4	$\gamma(OH_{A}\cdots N_{B})$, $\gamma(NH_{B}\cdots O_{A'})$, $\delta(OH_{A}\cdots N_{B})$, $\tau(OH_{A'}\cdots N_{B'})$
		17	0	8	$\gamma(OH_{A'}\cdots N_{B'})$, $\gamma(NH_{B'}\cdots O_{A})$
		13	1	5	$\gamma(OH_{A'}\cdots N_{B'})$, $\gamma(NH_{B'}\cdots O_{A})$
		10	0	2	$\delta(NH_{B}\cdots O_{A'})$, $\gamma(NH_{B'}\cdots O_{A})$, $\gamma(OH_{A}\cdots N_{B})$
		2	0	5	$\gamma(OH_{A'}\cdots N_{B'})$, $\gamma(OH_{A}\cdots N_{B})$, $\gamma(NH_{B'}\cdots O_{A})$

Abbreviations: ov, overlapped; ν, stretching; δ, in-plane bending; γ, out-of-plane bending; τ, torsion; 5R, five-membered pyrrole ring; 6R, six-membered pyridine ring.
A and B denote the two symmetry-independent molecules present in the crystal structure of 5OH7AI, while A′ and B′ correspond to their symmetry-related counterparts included to reproduce the experimental hydrogen-bonding network.
Subscripts: HB, hydrogen atom involved in hydrogen bonding; F, hydrogen atom not involved in hydrogen bonding. For hydroxyl and amino groups, these refer to the H atom itself, while for C–O groups (e.g. in C–OH), the notation indicates whether the O–H hydrogen is involved (HB) or free (F).
[a] Calculated frequencies were scaled. Details are given in Section 3.4.
[b] Assignments obtained from PED analysis calculated by FCART07 and verified using the Chemcraft program.

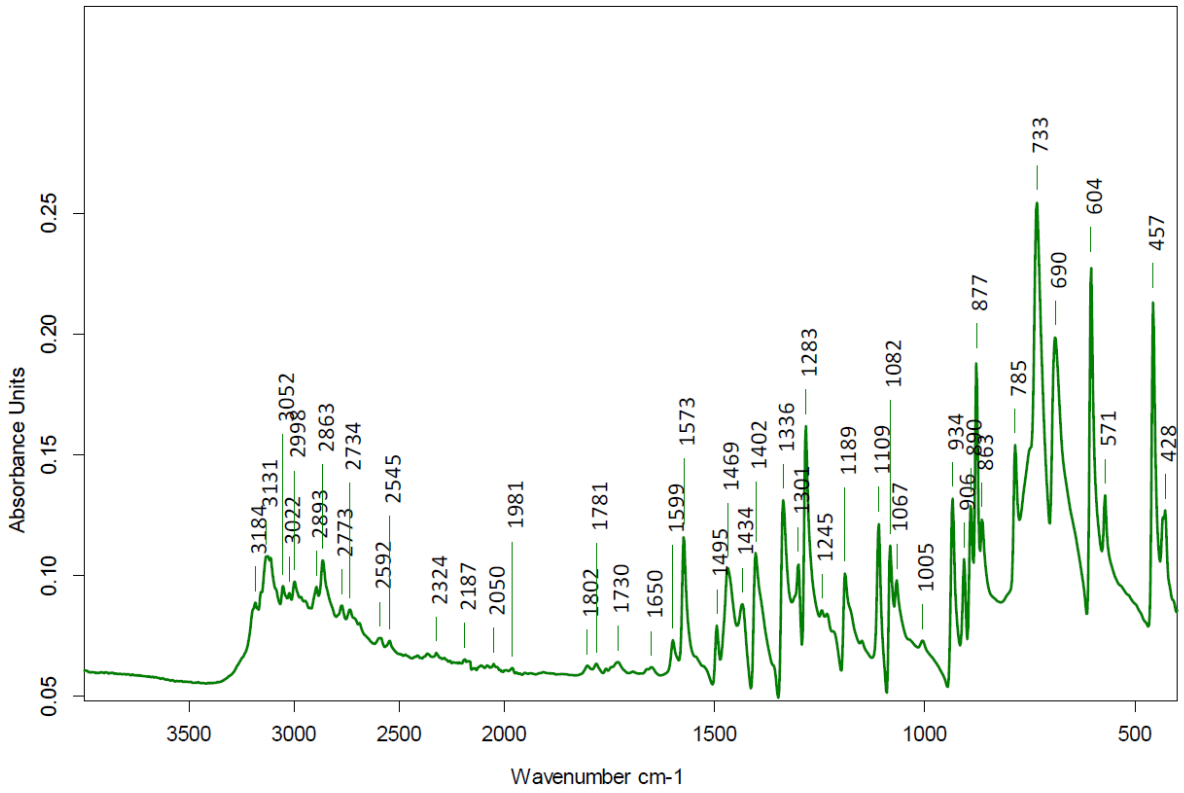

Figure S5. Original FT-IR spectrum of 5Cl7AI in the range from 4000 cm^{-1} to 400 cm^{-1}.

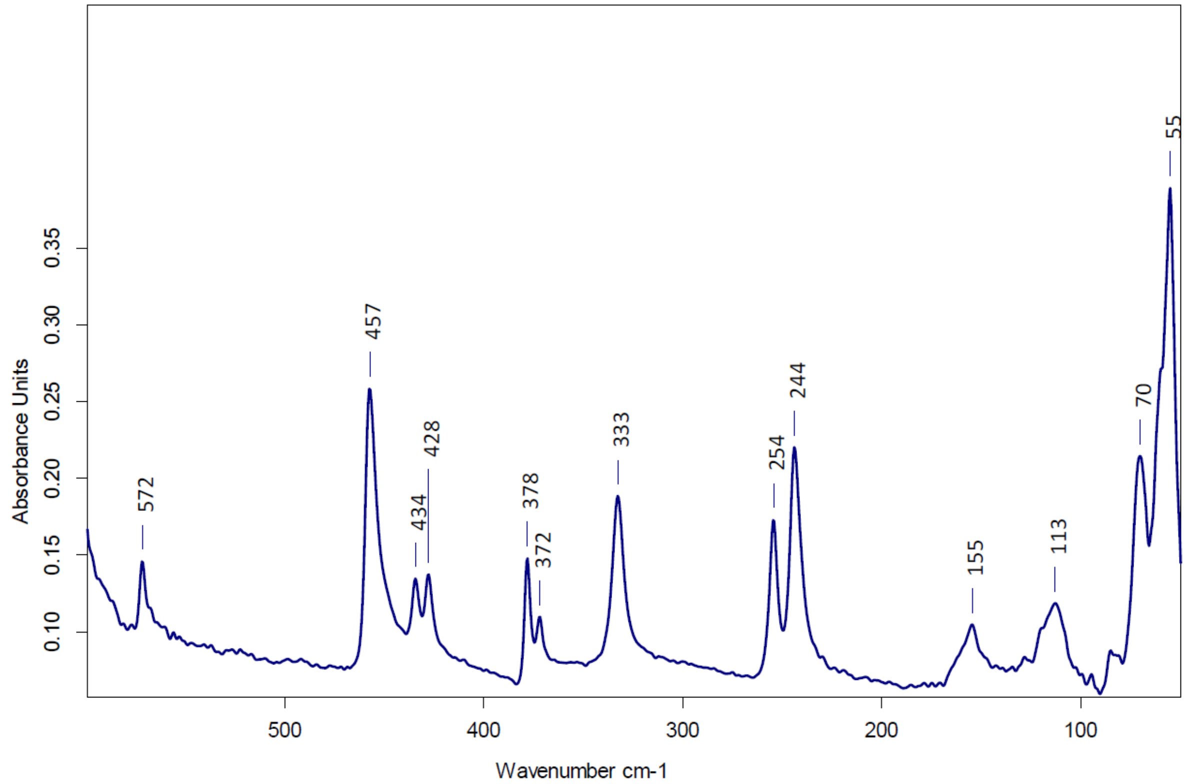

Figure S6. Original FT-IR spectrum of 5Cl7AI in the range from 600 cm^{-1} to 50 cm^{-1}.

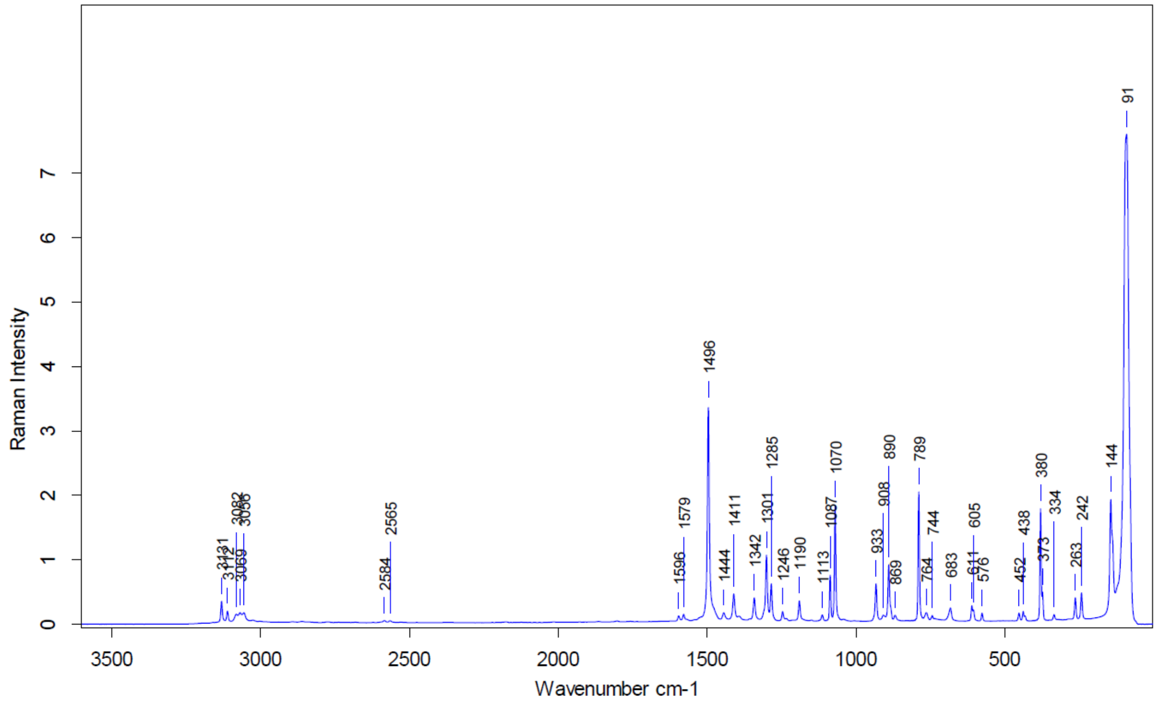

Figure S7. Original FT-Raman spectrum of 5Cl7AI in the range from 3600 cm^{-1} to 50 cm^{-1}.

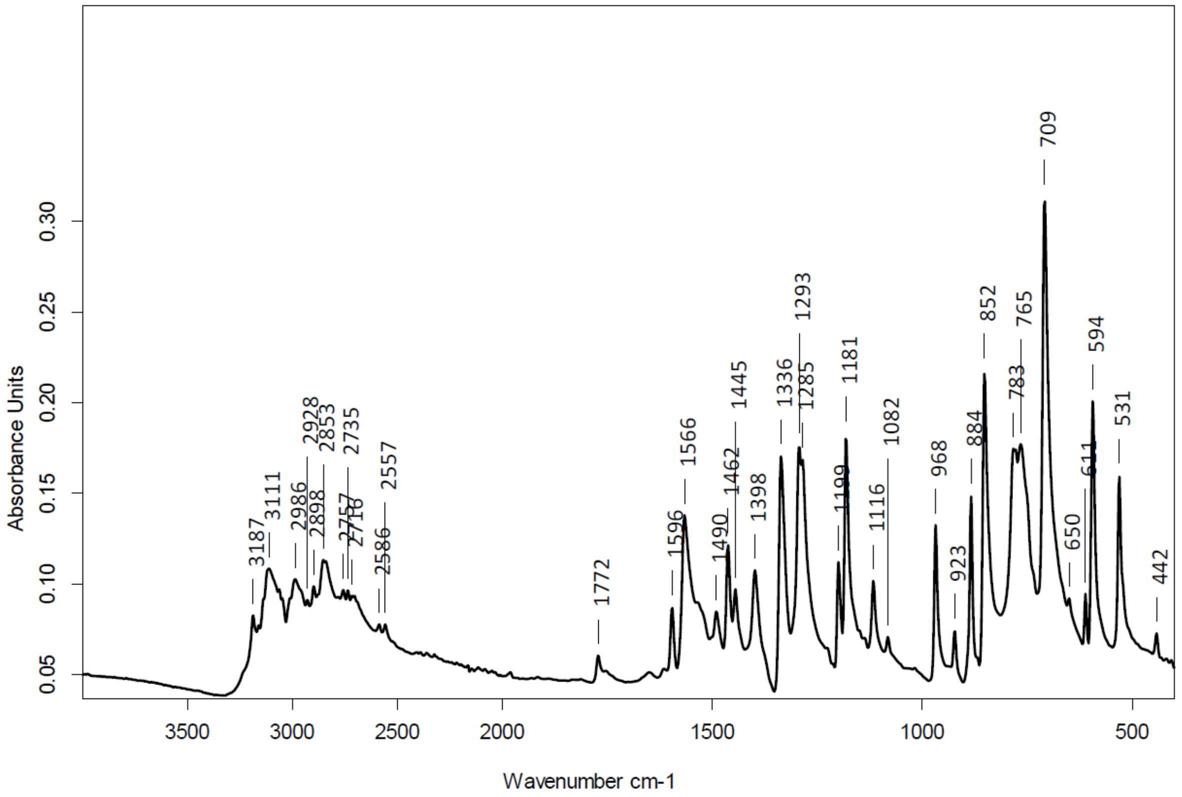

Figure S8. Original FT-IR spectrum of 4,5Cl7AI in the range from 4000 cm^{-1} to 400 cm^{-1}.

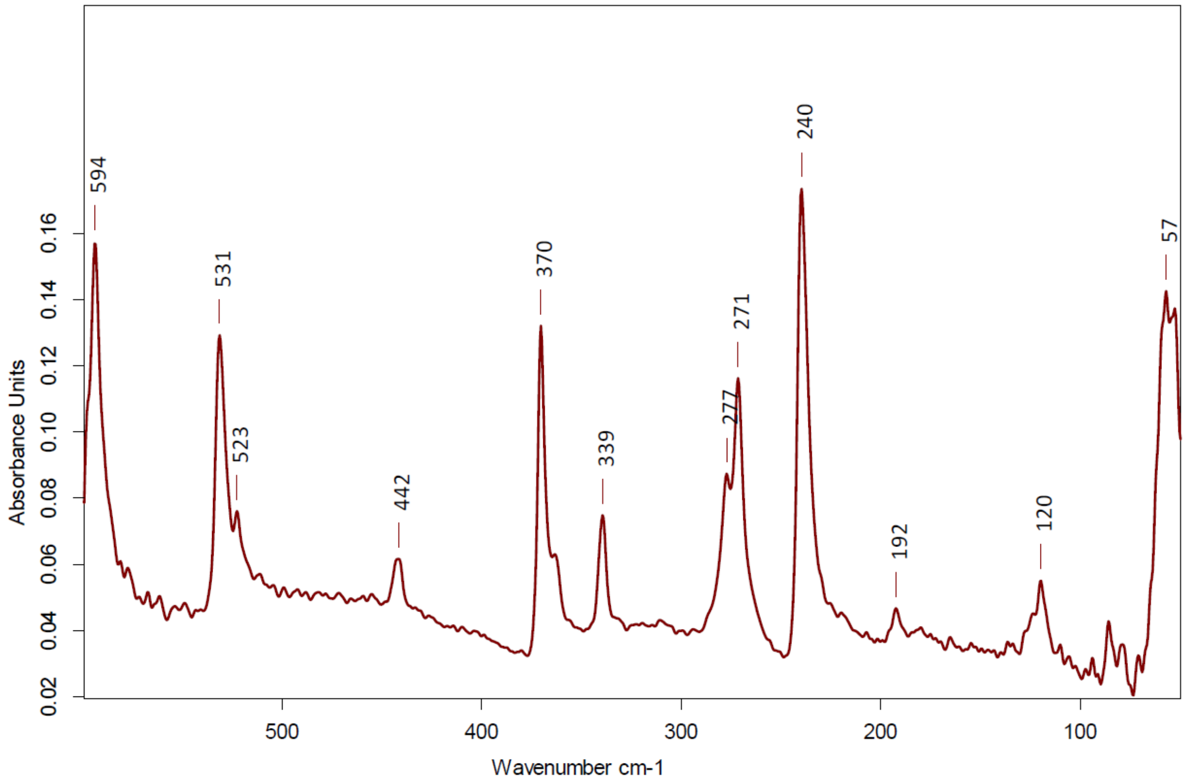

Figure S9. Original FT-IR spectrum of 4,5Cl7AI in the range from 600 cm^{-1} to 50 cm^{-1}.

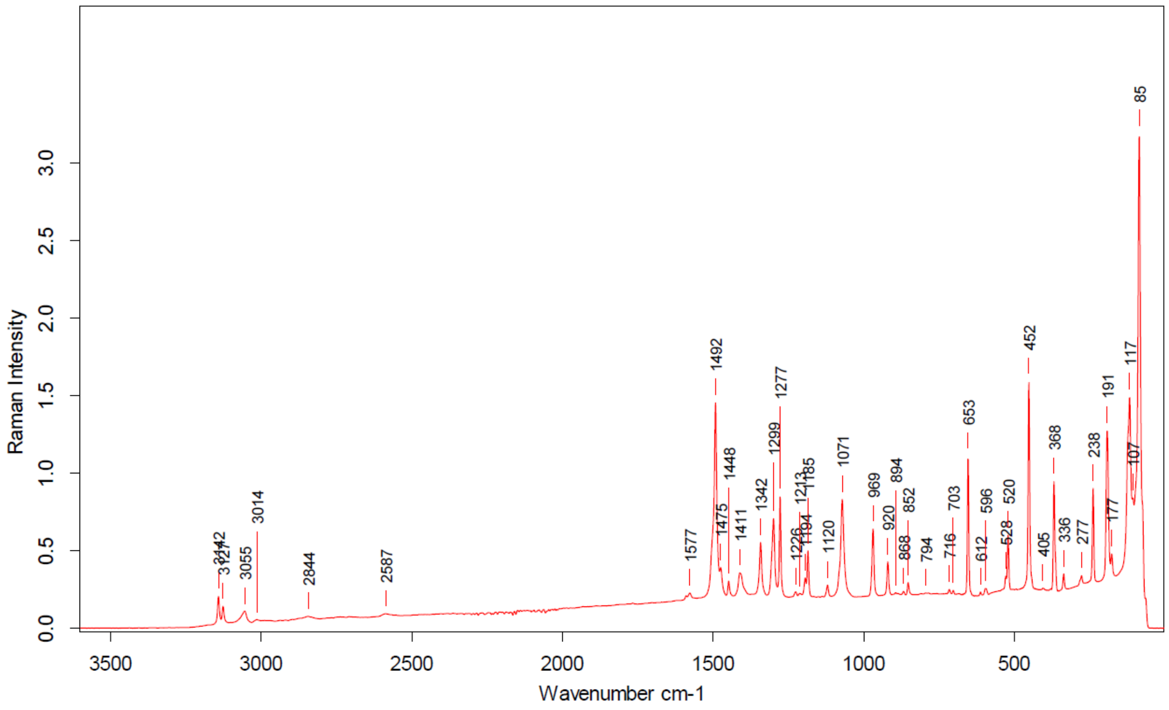

Figure S10. Original FT-Raman spectrum of 4,5Cl7AI in the range from 3600 cm^{-1} to 50 cm^{-1}.

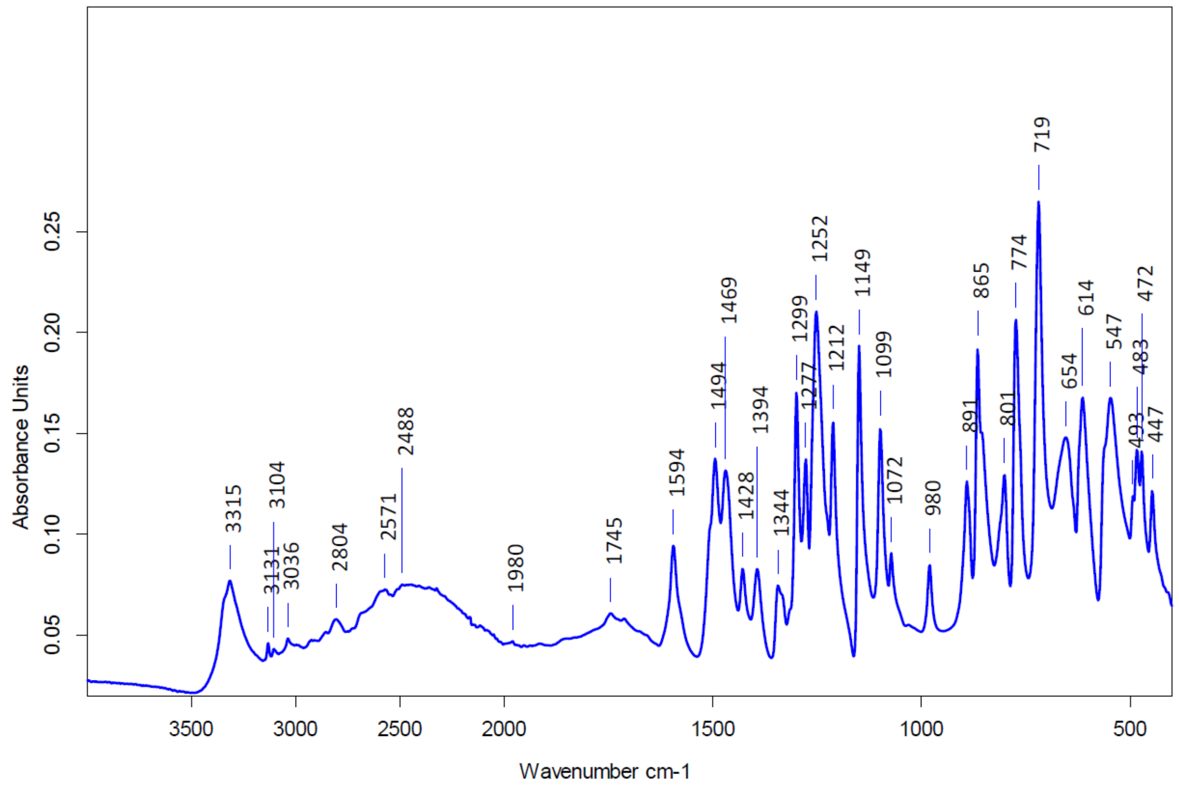

Figure S11. Original FT-IR spectrum of 5OH7AI in the range from 4000 cm^{-1} to 400 cm^{-1}.

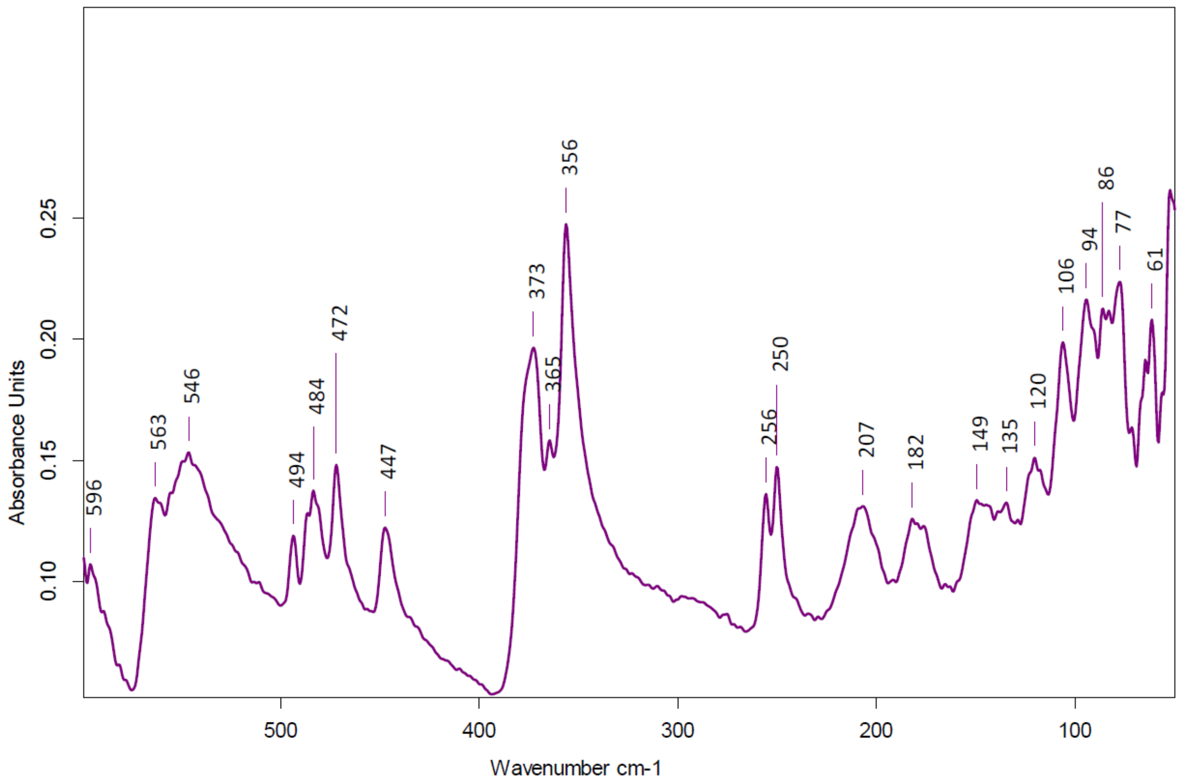

Figure S12. Original FT-IR spectrum of 5OH7AI in the range from 600 cm^{-1} to 50 cm^{-1}.

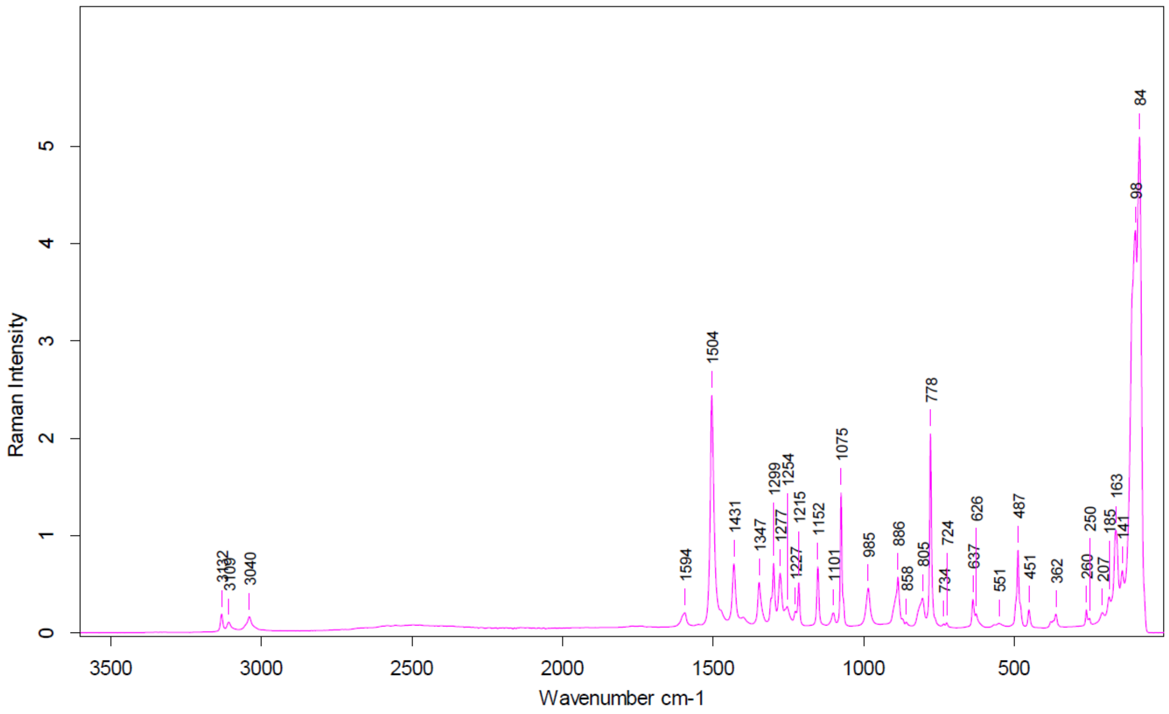

Figure S13. Original FT-Raman spectrum of 5OH7AI in the range from 3600 cm^{-1} to 50 cm^{-1}.

Symmetry codes for the molecular models of 5Cl7AI:

17	-5.354731000	-0.675835000	2.418414000
7	0.205822000	0.702902000	1.698496000
1	0.832424000	0.583559000	0.890444000
6	0.525556000	1.256805000	2.914668000
1	1.525756000	1.622489000	3.096712000
6	-0.559370000	1.250251000	3.750563000
1	-0.588678000	1.623737000	4.763078000
6	-1.630535000	0.653300000	3.011384000
6	-2.971131000	0.345188000	3.249197000
6	-3.671486000	-0.253625000	2.213522000
6	-3.054794000	-0.534261000	0.985346000
1	-3.623735000	-1.003877000	0.188918000
7	-1.778675000	-0.250104000	0.734660000
6	-1.105427000	0.326545000	1.732686000
17	5.354731000	0.675835000	-2.418414000
7	-0.205822000	-0.702902000	-1.698496000
1	-0.832424000	-0.583559000	-0.890444000
6	-0.525556000	-1.256805000	-2.914668000
1	-1.525756000	-1.622489000	-3.096712000
6	0.559370000	-1.250251000	-3.750563000
1	0.588678000	-1.623737000	-4.763078000
6	1.630535000	-0.653300000	-3.011384000
6	2.971131000	-0.345188000	-3.249197000
6	3.671486000	0.253625000	-2.213522000
6	3.054794000	0.534261000	-0.985346000
1	3.623735000	1.003877000	-0.188918000
7	1.778675000	0.250104000	-0.734660000
6	1.105427000	-0.326545000	-1.732686000
1	-3.453357000	0.559729000	4.196818000
1	3.453357000	-0.559729000	-4.196818000

Symmetry codes for the molecular models of 4,5Cl7AI:

17	-3.682987000	0.713601000	4.771737000
17	-5.355028000	-0.686643000	2.382983000
7	0.207238000	0.705507000	1.702091000
1	0.833364000	0.585398000	0.893830000
6	0.525848000	1.259973000	2.917906000
1	1.525296000	1.626134000	3.101944000
6	-0.559983000	1.253686000	3.752656000
1	-0.598726000	1.625161000	4.764906000
6	-1.624908000	0.656877000	3.010110000
6	-2.966356000	0.347081000	3.242538000
6	-3.681675000	-0.255453000	2.211026000
6	-3.047524000	-0.530256000	0.987939000
1	-3.620336000	-1.000399000	0.194729000
7	-1.774435000	-0.248885000	0.733478000
6	-1.102790000	0.328433000	1.732632000
17	3.682987000	-0.713601000	-4.771737000
17	5.355028000	0.686643000	-2.382983000
7	-0.207238000	-0.705507000	-1.702091000
1	-0.833364000	-0.585398000	-0.893830000
6	-0.525848000	-1.259973000	-2.917906000
1	-1.525296000	-1.626134000	-3.101944000
6	0.559983000	-1.253686000	-3.752656000
1	0.598726000	-1.625161000	-4.764906000
6	1.624908000	-0.656877000	-3.010110000
6	2.966356000	-0.347081000	-3.242538000
6	3.681675000	0.255453000	-2.211026000
6	3.047524000	0.530256000	-0.987939000
1	3.620336000	1.000399000	-0.194729000
7	1.774435000	0.248885000	-0.733478000
6	1.102790000	-0.328433000	-1.732632000

Symmetry codes for the molecular models of 5OH7AI:

8	-0.070589000	0.844322000	-0.867607000
1	-0.745350000	0.238948000	-0.425879000
7	-1.411079000	6.094180000	-0.600581000
1	-1.238016000	6.845206000	-1.250074000
7	-0.362049000	4.324232000	-1.860994000
6	-2.074958000	6.183491000	0.607589000
1	-2.463188000	7.131565000	0.951174000
6	-2.126362000	4.945726000	1.205751000
1	-2.583672000	4.721021000	2.158754000
6	-1.460446000	4.029832000	0.324621000
6	-1.151515000	2.661657000	0.334325000
1	-1.424352000	2.014622000	1.161439000
6	-0.467435000	2.156160000	-0.770642000
6	-0.103769000	3.012978000	-1.834314000
1	0.417561000	2.587727000	-2.688497000
6	-1.023342000	4.785145000	-0.801645000
8	6.955563000	-1.786103000	1.428229000
1	7.802361000	-1.347151000	1.277189000
7	2.742358000	1.066915000	-0.480075000
1	1.740821000	0.859148000	-0.530260000
7	3.534286000	-0.926271000	0.637183000
6	3.347029000	2.192669000	-0.997513000
1	2.744596000	2.965529000	-1.452511000
6	4.711848000	2.129846000	-0.819748000
1	5.427693000	2.879293000	-1.125007000
6	4.980849000	0.890625000	-0.155077000
6	6.117319000	0.215328000	0.312443000
1	7.113238000	0.638320000	0.200204000
6	5.929449000	-1.020670000	0.927921000
6	4.633570000	-1.554129000	1.072199000
1	4.500585000	-2.515164000	1.558923000
6	3.718385000	0.254316000	0.041157000
8	-5.518407000	-0.887132000	-0.827466000
1	-6.340147000	-1.100443000	-0.367204000
7	-1.209777000	-0.829783000	2.485752000
1	-0.226968000	-0.955479000	2.239010000
7	-2.092072000	-0.519841000	0.256679000
6	-1.759837000	-1.194293000	3.700043000
1	-1.124407000	-1.319513000	4.565126000
6	-3.123336000	-1.342213000	3.587233000
1	-3.803853000	-1.597942000	4.386652000
6	-3.449404000	-1.100243000	2.211675000
6	-4.608569000	-1.126592000	1.423029000
1	-5.581389000	-1.354554000	1.852930000
6	-4.469653000	-0.870976000	0.060417000
6	-3.205313000	-0.567443000	-0.481777000
1	-3.110095000	-0.355108000	-1.541836000
6	-2.224518000	-0.794017000	1.555082000
8	1.175186000	-2.038618000	1.369390000
1	2.024908000	-1.610690000	1.049873000
7	-2.012421000	-3.998506000	-2.550535000
1	-2.819112000	-4.599709000	-2.492293000
7	-1.439005000	-3.992761000	-0.205756000
6	-1.509303000	-3.398924000	-3.688965000
1	-1.977958000	-3.563191000	-4.648649000

6	-0.410096000	-2.635692000	-3.367636000
1	0.180510000	-2.052017000	-4.059221000
6	-0.217236000	-2.759294000	-1.951210000
6	0.666893000	-2.224874000	-1.002716000
1	1.453881000	-1.533930000	-1.283504000
6	0.461939000	-2.579924000	0.328406000
6	-0.579485000	-3.472952000	0.673538000
1	-0.711959000	-3.739364000	1.718798000
6	-1.242937000	-3.619541000	-1.468724000
